# Discovery of Potent Glycosidases Enables Quantification of Smoke-Derived Phenolic Glycosides through Enzymatic Hydrolysis

**DOI:** 10.1021/acs.jafc.4c01247

**Published:** 2024-05-10

**Authors:** Youtian Cui, Mary Riley, Marcus V. Moreno, Mateo M. Cepeda, Ignacio Arias Perez, Yan Wen, Lik Xian Lim, Eric Andre, An Nguyen, Cody Liu, Larry Lerno, Patrick K. Nichols, Harold Schmitz, Ilias Tagkopoulos, James A. Kennedy, Anita Oberholster, Justin B. Siegel

**Affiliations:** † Genome Center, University of California, Davis, California 95616, United States; ‡ Microbiology Graduate Group, University of California, Davis, California 95616, United States; § Department of Chemistry, University of California, Davis, California 95616, United States; ∥ Department of Viticulture & Enology, University of California, Davis, California 95616, United States; ⊥ Department of Food Science & Technology, University of California, Davis, California 95616, United States; # UC Davis Coffee Center, University of California, Davis, California 95616, United States; ∇ Food Safety and Measurement Facility, University of California, Davis, California 95616, United States; ○ VinZymes, LLC, Davis, California 95616, United States; ◆ March Capital US, LLC, Davis, California 95616, United States; ¶ T.O.P., LLC, Davis, California 95616, United States; †† Graduate School of Management, University of California, Davis, California 95616, United States; ‡‡ Department of Computer Science, USDA/NSF AI Institute for Next Generation Food Systems (AIFS), University of California, Davis, California 95616, United States; §§ PIPA, LLC, Davis, California 95616, United States; ∥∥ Department of Biochemistry and Molecular Medicine, University of California, Davis, California 95616, United States

**Keywords:** smoke taint, volatile phenols, glycosidase, volatile-phenol glycosides, hydrolysis

## Abstract

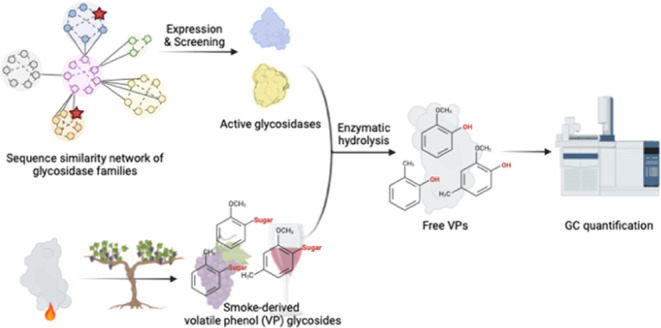

When grapes are exposed to wildfire smoke, certain smoke-related volatile phenols (VPs) can be absorbed into the fruit, where they can be then converted into volatile-phenol (VP) glycosides through glycosylation. These volatile-phenol glycosides can be particularly problematic from a winemaking standpoint as they can be hydrolyzed, releasing volatile phenols, which can contribute to smoke-related off-flavors. Current methods for quantitating these volatile-phenol glycosides present several challenges, including the requirement of expensive capital equipment, limited accuracy due to the molecular complexity of the glycosides, and the utilization of harsh reagents. To address these challenges, we proposed an enzymatic hydrolysis method enabled by a tailored enzyme cocktail of novel glycosidases discovered through genome mining, and the generated VPs from VP glycosides can be quantitated by gas chromatography–mass spectrometry (GC–MS). The enzyme cocktails displayed high activities and a broad substrate scope when using commercially available VP glycosides as the substrates for testing. When evaluated in an industrially relevant matrix of Cabernet Sauvignon wine and grapes, this enzymatic cocktail consistently achieved a comparable efficacy of acid hydrolysis. The proposed method offers a simple, safe, and affordable option for smoke taint analysis.

## 1 Introduction

Many wine regions such as Australia, North America, South America, and Europe are periodically ravaged by devastating wildfires, seemingly exacerbated by prolonged droughts, intense heatwaves, and years of uncontrolled forest growth.^1,2^ These fires have significant detrimental impacts on wines produced from smoke-exposed fruit as a consequence of the imparting of negative smoke aromas and flavors to the wine.^1,3,4^ This “smoke taint” occurs when grape vines exposed to wildfire smoke absorb the volatile phenols (VPs) produced from lignin combustion products. Wines produced from these smoke-exposed grapes acquire undesirable smoky aromas, often described as ’burnt wood’, ’ashtray’, ’burning rubber’, and ’smoked meat’.^5^ These persistent aromas and flavors can be sufficiently high in concentration that resultant wines are considered unmarketable.

**Figure 1:**
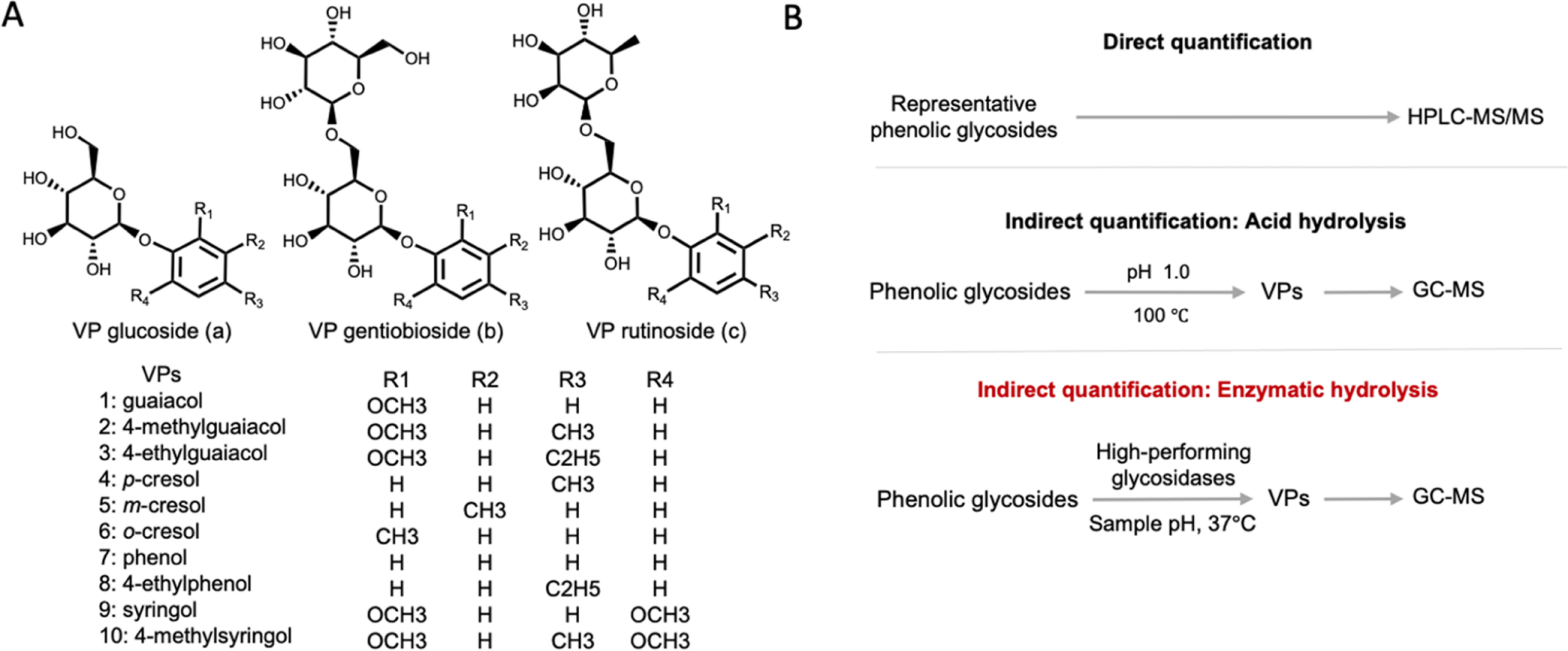
Structures and the quantification methods of smoke-associated volatile-phenol glycosides. (A) The structures of bound VP glycosides as the smoke taint markers. (B) VP glycosides were used to assess the level of smoke impact. Available methods can be categorized into direct quantification and indirect quantification.

Many VPs including guaiacol **1**, 4-methylguaiacol **2**, 4-ethylguaiacol **3**, cresols (*p*- **4**, *m***- 5**, *o*- **6**), phenol **7**, 4-ethylphenol **8**, syringol **9**, and 4-methylsyringol **10** have been identified as smoke taint markers.^4,5^ These VPs can be absorbed by the grape berries and sequentially metabolized into their related nonvolatile glycoconjugates (volatile-phenol glycosides), often referred to as “bound (-form) VPs” (Figure 1A). As a result, VPs are largely accumulated in the grape tissue as VP glycosides, such as monoglucosides **a** (phenolic β-d-glucopyranoside), gentiobiosides **b**, and rutinosides **c** (Figure 1A).^6,7^ Both free and bound VPs play a role in the perception of smoke taint in wine. The odorless VP glycosides can be converted into free, odor-active VPs via yeast metabolism during fermentation, the aging process, or through enzymes in saliva, hence releasing the smoke taint aroma upon consumption.^5,6,8−10^ Given the established correlation between the presence of free VPs and their corresponding glycosides in wine and the perceived smoke flavor, it is crucial to accurately quantify both free and bound VPs.

In the analysis of free VPs, gas chromatography–mass spectrometry (GC–MS) has been the standard analytical technique (Figure 1B).^11^ It is typically implemented following a variety of sample preparation procedures, including liquid–liquid extraction (LLE) or stir bar sorptive extraction,^12^ and headspace solid-phase microextraction (HS-SPME).^9,13−16^ GC–MS/MS can serve as a more sensitive and specific option for the quantitation of free VPs. However, GC–MS/MS instrumentation is more complex and expensive than GC–MS instrumentation, which limits its accessibility and widespread adoption in many laboratories.

Multiple analytical methods have been developed to measure bound VPs (Figure 1B). Direct quantitation by liquid chromatography tandem mass spectrometry (LC–MS/MS) has become widely accepted.^17^ However, because this quantitation process requires calibration from standards, it can be used only for commercially available glycosides. Given the limited range of phenolic glycosides that can be purchasable, it remains uncertain whether these glycosides can accurately represent the complete glycoside profile in smoke-impacted grapes and wine. Additionally, adopting LC–MS/MS poses challenges for many laboratories due to the equipment cost, the need for professional training, the time for sample preparation by solid-phase extraction (SPE) extraction, and the limited number of samples that can be processed each day because of the method’s relatively lengthy run time.

Indirect quantification methods in which free VPs are released from bound VPs, making them available for detection using GC–MS have gained interest as a simpler option than measuring the individual VP glycosides directly (Figure 1B).^18−20^ The glycosidic bonds between the VPs and their glycosidically bound sugars can be cleaved by hydrolysis. Acid hydrolysis is the predominant hydrolysis method used and involves the use of strong acid (pH 1.0) with a 1–4 h treatment at a temperature of 100 °C.^18^ Despite its simplicity, it is still imperative to explore alternative methods due to several issues. First, achieving significant VP release from their bound form requires maintaining a stringent pH of 1.0. Dealing with such a high concentration of strong acid and preserving a pH of 1.0 can be difficult and require vigilant attention. Second, the recovery rate of different VPs varies, and the strong acid may lead to aglycone degradation and underestimation of such acid-labile VPs.^20^ Third, the method is sensitive to the experimental conditions. Enzymatic hydrolysis, using glucosidase enzyme not specifically intended for this purpose, has been proposed and tested as an alternative.^5^ However, enzymatic hydrolysis using these commercially available enzymes was reported to be less effective when compared to acid hydrolysis.^5^

In this study, high-performing glycosidases, *Cb*GglB-1 and *Aory*Rut, were identified for targeted VP glycosides (Figure 1A) through genome mining. These enzymes were primarily screened using LC–MS against high concentrations of VP glycosides. The application of *Cb*GglB-1 and *Aory*Rut facilitated the rapid and high-yield cleavage of glycosidic bonds, thereby liberating VPs that could be sequentially quantified by HS-SPME GC–MS. By examination of the same sample before and after enzymatic hydrolysis, free VPs and the total VPs following their release from VP glycosides can be quantitated. The observed difference indicated the amount of VP glycosides. The enzyme’s activity was further evidenced by spiking low levels of VP glycosides as the substrate in wine, with LC–MS/MS analysis subsequently revealing their extremely low or near nondetectable levels. The efficiency of enzymatic hydrolysis was found to be nearly equivalent to that of acid hydrolysis in processing both high- and low-smoke-impacted wines and grape berries. The enzymatic hydrolysis method, which does not require harsh conditions or chemicals, appears to be a cost-effective and sustainable method for commercial samples. It can be easily adopted and scaled in commercial lab setups.

## 2 Materials and Methods

### 2.1 Bacterial Strains, Plasmids, and Chemical Reagents

The pET29 (+b) plasmids containing the protein-encoding genes were expressed in the *Escherichia coli* BLR (DE3). All genes were purchased from Twist Biosciences as synthetic genes optimized for *E. coli* codon usage with the infusion of 6-histidine at the C-terminus. The sequences of genes encoding all glycosidases in the present work are listed in Table S8.

Analytical grade chemicals and high-performance liquid chromatography (HPLC) grade solvents were purchased from Sigma-Aldrich and Merck (Darmstadt, Germany). All VP standards and VP glycosides were purchased from Toronto Research Chemicals (Toronto, Canada), C/D/N Isotopes Inc. (Quebec, Canada), and EPTES (Vevey, Switzerland). The chemicals were prepared in 100% ethanol at 100 mg/L as the original stock and dissolved in a Milli-Q water solution at 5 mg/L for each use. The stocks were stored at −20 °C. Cobalt slurry for protein purification was purchased from Thermo Fisher Scientific.

### 2.2 Grape and Wine Samples

The heavily smoke-tainted wines were made from 2020 Cabernet Sauvignon grapes from the Dry Creek Valley AVA (American Vineyard Area) in Sonoma County. The grapes were exposed to the LNU Lightening Complex wildfire smoke and were harvested on October 1st, 2020. The grapes were processed at the UC Davis LEED Platinum teaching and research winery using standards experimental winemaking protocols as described in previous research.^21^

The smoke-exposed grapes were 2020 Cabernet Sauvignon harvested from a vineyard in St. Helena AVA, Napa County on October 8th, 2020. These grapes were exposed to the LNU Lightening Complex as well as to Glass wildfire smoke. The no-to-low-smoke (baseline) Cabernet Sauvignon wine was made from grapes from the student vineyards at the Robert Mondavi Institute at UC Davis. The grapes were harvested on September 16th, 2020, and processed at the UC Davis LEED Platinum teaching and research winery using standards experimental winemaking protocols.^21^

The chemical parameters of the wines were analyzed on each testing day of the descriptive analysis. The titratable acidity (TA) was measured using a Mettler-Toledo DL50 titrator (Mettler-Toledo Inc., Columbus, OH); pH was measured using an Orion 5-star pH meter (Thermo Fisher Scientific, Waltham, MA); alcohol content % (v/v) was measured using an alcohol analyzer (Anton Parr, Ashland, VA); acetic acid, malic acid, and residual sugar (RS) were determined by enzymatic analysis using a Gallery automated analyzer (Thermo Fisher Scientific, Waltham, MA).

### 2.3 SSN and Sequence Analysis

We followed the procedures of constructing SSNs as outlined in a previous publication.^22^ Briefly, the SSN construction involves three key steps: First, collecting input sequences; second, analyzing these sequences for phylogenetic information using EFI-EST; and third, visualizing the results. The Interpro IPR001360 collection of GH1 enzyme sequences combined with JGI IMG Integrated Microbial Genomes & Microbiomes database annotated GH1 enzymes was used as the input for EFI-EST analysis of GH1 while Interpro IPR001547 annotated as rutinosidase was used as the input for GH5. For both networks, only Ref50 clusters were used. A sequence identity threshold of 45 was used as the parameter for filtering the sequences into clusters in SSN, and representative node networks with 70% identity were displayed by Cytoscape.^23^

### 2.4 Protein Expression and Purification

*E. coli* was first grown overnight as the starter culture at 37 °C in Terrific Broth medium (1% tryptone, 0.5% yeast extract, 0.5% NaCl,) supplemented with kanamycin (50 μg/mL final concentration) and MgSO_4_ (1 mM final concentration). The culture for protein expression was diluted by ∼50-fold to 500 mL from the starter culture. Cells then grew until OD_600_ reached ∼0.6 at 37 °C, and IPTG was supplemented to a final concentration of 0.5 mM for induction at 16 °C for 24 h. Cells were centrifuged (4700*g*, 4 °C, 10 min), resuspended in 40 mL of lysis buffer (50 mM HEPES, pH 7.0, 300 mM NaCl, 10% glycerol, 1 mM MgSO_4_, 15 mM imidazole), and sonicated for 2 min at 4 °C. Lysed cells were centrifuged at 4700*g* at 4 °C for 30 min to remove cell debris. The supernatant was loaded on a gravity flow column with 1 mL of cobalt slurry (Thermo Fisher Scientific, CAT# PI-90091), which was prebalanced with 30 mL of wash buffer (50 mM HEPES, pH 7.0, 300 mM NaCl, 10% glycerol, 1 mM MgSO4, 15 mM imidazole). The cobalt resin was then washed 3 times with 10 mL of wash buffer; proteins were eluted with 0.6 mL of elution buffer (50 mM HEPES, pH 7.0, 300 mM NaCl, 10% glycerol, 1 mM MgSO_4_, 1 mM TCEP, 200 mM imidazole). Protein samples were immediately buffer exchanged with spin concentrators (Satorius, CAT# VS0112) into storage buffer (50 mM HEPES, pH 7.0, 300 mM NaCl, 10% glycerol, 1 mM MgSO_4_) and stored at 4 °C until activity characterization. Protein concentrations were determined by measuring the absorbance at 280 nm. The protein samples were further analyzed by 12% SDS-PAGE gel (Figure S6).

### 2.5 Initial Activity Screening by Liquid Chromatography–Mass Spectrometry (LC–MS)

Purified enzymes with a calibrated concentration of about 0.1 mg/mL were added into both acetic acid buffer at pH 3.5 and baseline wine samples with 4.5 mg/L of substrates guaiacol glucoside **1a**, guaiacol gentiobioside **1b**, and 4-methylguaiacol rutinoside **2c** (Figure 1A). The reaction mixture was kept at 37 °C for 24 or 4 h. After cooling on ice, the reactions were quenched by addition of 50% volume of acetonitrile and then centrifuged. Proteins in the reactions were denatured and precipitated, and the supernatant that contained glycosides was subjected to LC–MS analysis.

**Figure 2:**
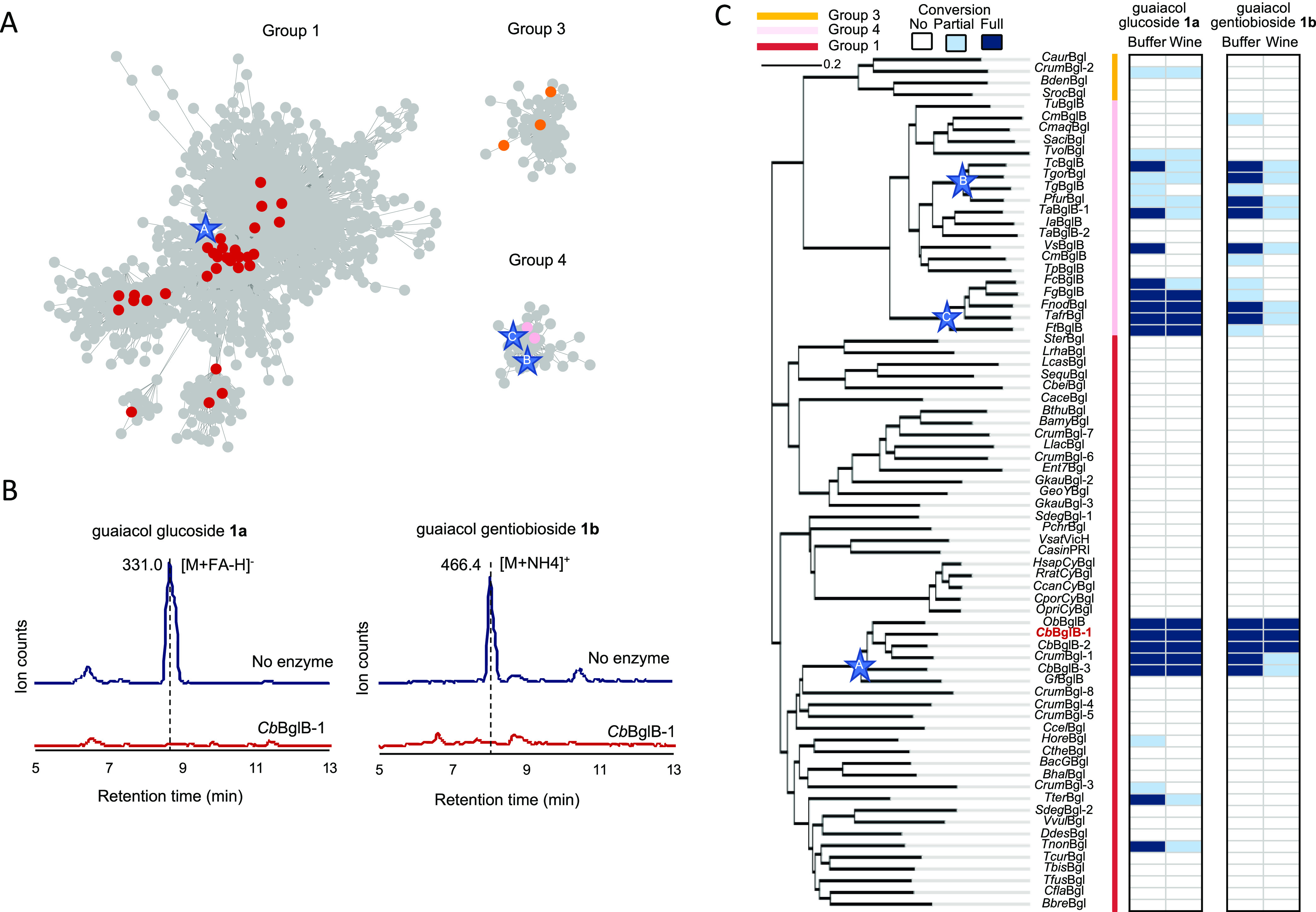
Initial screening of active glycosidases (GHs) on guaiacol glucoside **1a** and guaiacol gentiobioside **1b** through genome mining. (A) Sequence similarity network (SSN) of GH1 enzyme family. Only the groups containing the tested sequences are depicted in the figure, while the complete SSN can be found in the Supporting Information. The most active GHs located in representative nodes A in group 1 and B, C in group 4. (B) Utilization of LC–MS analysis for activity screening in wine. This figure demonstrates the application of this method using *Cb*BglB-1 as a representative example. The semiquantitative activity could be evaluated by comparing total ion counts in MS between samples with the added enzyme and those without it. (C) Semiquantitative heatmap of the degrees of conversion by GH1 enzymes on **1a** and **1b** in buffer and wine. Candidates such as *Cb*BglB-1 were mixed with baseline wine which had been fortified with 4.5 mg/L each of compounds **1a** and **1b**. The reaction was at 37 °C for 4 h duration. The high-activity clusters corresponding to the SSN are also labeled as stars. The most promising candidate *Cb*BglB-1 is highlighted in red.

Reverse-phase high-performance liquid chromatography and mass spectrometry (LC–MS) for analysis were carried out using Agilent 1260 series instruments with a Poroshell 120 EC-C18 (Agilent, 4.6 × 50 mm^2^, 2.7 μm) column. Mass spectrometry was carried out using an Agilent 6120 single quadrupole spectrometer with electrospray ionization (ESI) in either positive-ion mode or negative-ion mode. The gas temperature was 350 °C, the drying flow was 13.0 L/min, and the capillary voltage was 4300 V. Each sample was analyzed in triplicate. The mobile phase consisted of three periods: 70% H_2_O with 0.1% formic acid and 30% acetonitrile (ACN) with 0.1% formic acid constant for 0–5 min; linear gradient to 10% H_2_O with 0.1% formic acid and 90% ACN with 0.1% formic acid from 8 to 19 min; directly jumping to 70% H_2_O with 0.1% formic acid and 30% ACN with 0.1% formic acid and lasting for 19–25 min. The HPLC flow rate was 0.5 mL/min, and the injection volume was 3 μL. The parameters of the mass spectrum were adjusted accordingly for different glycosides as shown in Figures 2 and 4. The compounds were identified by comparing their retention times and *m*/*z* values with those of the glycoside standards, and they were semiquantitative by comparing their peak areas to those of the glycoside standards. Agilent MassHunter (version 8.07.00) for qualitative analysis facilitated the isolation of target ion adducts identified by HPLC–MS.

### 2.6 Quantitation of Glycosidic Precursors by Liquid Chromatography Triple Quadrupole Mass Spectrometry (LC–MS/MS)

The concentrations of volatile phenol glycosidic precursors were determined by using solid-phase extraction (SPE) followed by liquid chromatography tandem mass spectrometry (LC–MS/MS) analysis as described in a previous publication.^24^ Liquid chromatography was performed on an Agilent 1290 Infinity UHPLC (Agilent Technologies, Santa Clara, CA) equipped with a binary pump, temperature-controlled autosampler and thermostated column compartment. An Agilent Poroshell Bonus-RP (150 mm × 2.1 mm, 2.7 μm) fitted with a matching guard column was used for chromatographic separation and was maintained at 40 °C. Mobile phase A was water with 10 mM ammonium formate, and mobile phase B was methanol/acetonitrile (1:1) with 10 mM ammonium formate. The gradient used for the separation was as follows: 0 min, 8% B; 1 min, 8% B; 6.5 min, 24.5% B; 7.5 min, 90% B; 9 min, 90% B; and 10 min, 8% B with a flow rate of 0.42 mL/min. The injection volume was 12 μL, and the column was equilibrated for 2 min before each injection.

Tandem mass spectrometry was performed on an Agilent 6460 triple quadrupole mass spectrometer (Agilent Technologies, Santa Clara, CA) with an Agilent JetStream electrospray source. Source conditions were sheath gas temperature 375 °C, sheath gas flow 11 L/min, drying gas temperature 250 °C, drying gas flow 12 L/min, nebulizer pressure 45 psi, capillary voltage 3500 V, and nozzle voltage 0 V. Detection of the glycosides was done using dynamic MRM. MRM transitions were determined and optimized using commercially available standards, and calibration curves were performed for all available glycosides with the linear range 0–500 ng/L. The retention times, MRM transitions, and other parameters are listed in Table S1.

### 2.7 Acid Hydrolysis and Enzymatic Hydrolysis

#### 2.7.1 Sample Preparation for Grape Berries

Samples were removed from the freezer; then, 65 g of berries were separated from cluster rachi, taking care to prevent berry cap and other nonberry debris from introduction into the sample container. Samples were thawed for 15–20 min at room temperature. 15 mL of water was added to the sample, homogenized with a high-speed commercial blender (Blendtec Classic 575 blender with four-side jar) for 1 min, paused for 1 min, and then homogenized for a further 30 s.

#### 2.7.2 Sample Preparation for the VP Glycoside Spike-Recovery Test

Eight commercially available β-d-glycosides including guaiacol glucoside **1a**, guaiacol gentiobioside **1b**, guaiacol rutinoside **1c**, 4-methylguaiacol rutinoside **2c**, *p*-cresol rutinoside **4c**, phenol rutinoside **7c**, syringol gentiobioside **9b**, and 4-methylsyringol gentiobioside **10b** were spiked in wine and berry homogenate (Figure 1A). Compounds **1a**, **1b**, and **1c** were spiked and analyzed separately due to their shared aglycon guaiacol, while the remaining glycosides (**2c**, **4c**, **7c**, **9b**, and **10b**) were analyzed concurrently in the same samples due to their diverse aglycon structures that can be resolved by GC–MS directly. The samples with spiked VP glycosides were subjected to enzymatic hydrolysis and acid hydrolysis and then quantified by HS-SPME GC–MS.

#### 2.7.3 Enzymatic Hydrolysis

4 g of the homogenized berry sample or 4 mL of wine was transferred into 20 mL GC vials purchased from Agilent. 16 μL of ethanolic d3-guaiacol (5 mg/L) internal standard was added to the samples (final concentration of 20 μg/kg in berry homogenate or 20 μg/L in wine). Glycosidase enzymes were then added to the samples. For enzymatic hydrolysis of real-world samples, the final concentrations of 4 and 1 mg/mL of *Cb*GglB-1 and *Aory*Rut were added, respectively. The reactions were conducted at 37 °C for 4 h. Forty percent w/v of NaCl was then added to the samples to stop the reactions, and GC vials were capped for GC–MS analysis.

#### 2.7.4 Acid Hydrolysis

Samples were aliquoted into 20 mL glass tubes in 10 mL, and the pH was adjusted to 1.0 with 4 M HCl. The acid was dropwise added to the samples, and pH was monitored in real time by a pH meter to make sure the endpoint pH 1.0 was achieved. Samples were then fortified with 40 μL of an ethanolic d3-guaiacol (5 mg/L) internal standard. Samples were then transferred from the glass tubes to 17 mL Teflon tubes (SPI Supplies, 02044-AB) equipped with tightly fitted caps. Samples were incubated at 100 °C for 1 h and then cooled over ice for 10 min before aliquoting 4 mL of wine or 4 g of grape homogenate into GC vials. Then, 40% w/v of NaCl was added to each sample, and the GC vials were capped for GC–MS analysis.

### 2.8 Quantitative HS-SPME GC–MS Analysis

#### 2.8.1 HS-SPME

Smart SPME arrow 1.1 mm DVB/CarbonWR/PDMS (Agilent 5610–5861) was used by a PAL3 robotic autosampler for sample injections. The SPME headspace settings: predesorption time: 4 min and temperature: 250 °C. Sample incubation time: 4 min. Sample vial penetration depth: 35 mm. Inlet penetration depth: 40 mm. Inlet penetration speed: 100 mm/s. Sample vial penetration speed: 35 mm/s. Sample extraction time: 9 min and extraction temperature: 60 °C. Heatex stirrer speed: 1000 rpm and temperature: 40 °C. Sample desorption time: 3 min

#### 2.8.2 GC–MS

An Intuvo 9000 GC system and 5977B inert plus single quadrupole EI MSD were used. All samples in 20 mL GC–MS headspace vials were added with 1.6 g of NaCl. The GC–MS injection mode was splitless at 250 °C. GC has a constant flow of 1.2 mL/min helium gas. The GC column was J&W DB-HeavyWAX Intuvo GC column module, 30 m, 0.25 mm, 0.25 μm (122–7132-INT). The oven program was 120 °C (hold 1 min); 9 °C/min to 250 °C (hold 0 min); 250 °C/min to 280 °C (hold 0 min). The guard chip temperature was 200 °C, bus temperature 280 °C, and MSD transfer line 280 °C. Calibration curves for all compounds were used for quantification and established by using the response factors to the internal standard. Agilent MassHunter software (version 10.2) for quantitative analysis was utilized to quantitate VPs by using the stable isotope dilution method (Table S2). Calculation of recovery rates of free VPs from VP glycosides involved a mass balance analysis between the free VPs and their bound glycosides. The recovery rate is calculated as the ratio of the concentration of generated VP to the theoretically maximum concentration of VP achievable with 100% recovery.

### 2.9 Statistical Analysis

All experiments were independently carried out in triplicate. The differences between samples were evaluated by student’s *t* test in GraphPad (https://www.graphpad.com/quickcalcs/ttest1.cfm). The *p*-values <0.05 indicates a statistically significant difference.

## 3 Results and Discussion

### 3.1 Identification of Active Glycosidases on Guaiacol Glycosides through Genome Mining

To identify enzymes with the ability to cleave glycosidic bonds in bound VPs, we broadly explored the sequence space of the glycosidase 1 (GH1) enzyme family through genome mining in a gene sequence database such as Uniprot^25^ and NCBI Genebank.^26^ The approach involved collecting and characterizing an assortment of representatives from the gene sequence database that would capture a considerable amount of sequence diversity within the targeted enzyme family. This process has been proven to be successful in new biocatalyst discovery.^27,28^ GH 1s catalyze the hydrolysis of the glycosidic bonds that form either between two or more carbohydrate molecules or between a carbohydrate molecule and a noncarbohydrate entity. The GH1 enzyme family is widely distributed in archaea, eubacteria, and eukaryotes.^29^ We chose the GH1 family as the primary target because GH 1s have diverse substrate specificities on both conjugated sugars and aglycons. Recently, a comprehensive examination of the functional variety within this group of enzymes further validated GH1 substrate promiscuity and its suitability for industrial purposes.^30^

A total of approximately 80,000 genes presumably annotated as the GH1 family were visualized via a sequence similarity network (SSN)^22^ based on their phylogenetic relationships, in which all sequences sharing 75% or more identity were grouped into a single meta node (Rep node) (Figure S1). A set of 73 synthetic genes encoding naturally occurring proteins were procured (Figure 2A). The 73 genes were distributed within the clusters of group 1 (49/73), group 3 (4/73), and group 4 (20/73), ranked by the total number of genes represented, and the three groups accounted for more than 70% of sequences in the GH1 family. The collection of genes represents a considerable diversity in sequence space with an average identity of 30% with respect to each other.

Synthetic genes encoding the 73 proteins were purchased, cloned into a pET29b+ vector with a C-terminal 6x histidine tag, and overexpressed in *E. coli*. The corresponding proteins were purified by IMAC and analyzed by SDS-PAGE. The obtained enzymes underwent stepwise testing to evaluate the ability to release VPs, and the activity was semiquantitatively assessed based on the degree of substrate disappearance postreaction by LC–MS (Figure 2B). Guaiacol and its glycosides were seen as the major markers for smoke exposure and thus were chosen as the screening substrates. We commenced the initial proof-of-concept screening under acetic acid buffer conditions at pH 3.5 with 4.5 mg/L guaiacol glucoside **1a** as the substrate at 37 °C over a 24-h period (Figure S2). We started with **1a** as the substrate not only for its simplicity and single glycosidic linkage but also because it can be a product derived from di-, tri-, or even more complex sugar forms, making it a versatile choice for the study. 45/73 enzymes were found to be active toward **1a**, while the other 28 enzymes were either inactive or not expressed in a soluble form.

Next, the enzymes were tested against two substrates, guaiacol glucoside **1a** and guaiacol gentiobioside **1b**, under acetic acid buffer conditions at pH 3.5 (Figure S3) and not smoke-impacted, baseline Cabernet Sauvignon wine without pH adjustment (Figure 2B,C) using a 4 h incubation time. The enzyme activity in both buffer systems was compared because the chemicals in wines, especially in red wines, such as ethanol, glucose, tannins, and metals can likely inhibit GHs, and the side-by-side comparison can provide the necessary information to determine whether the lack of activity in wine was due to inhibition. For guaiacol glucoside **1a**, 22 enzymes exhibited glycosidase activity out of which 15 were capable of completely catalyzing the release of guaiacol in an acetic acid buffer (Figure 2C). As for guaiacol gentiobioside **1b**, 18 enzymes were active, with 12 of them being able to fully catalyze the liberation of guaiacol in an acetic acid buffer. It was noted that the activity is focused on the enzymes in Ref50 clusters (highlighted as stars) of A0A4P2Q3W9 in group 1 and P22498 and A0A1E3G457 in group 4.

Inhibition in Cabernet Sauvignon was clearly observed for both substrates. Among the 12 enzymes that can fully utilize **1a** in acetic acid buffer, nine enzymes maintained complete functionality. However, in the case of **1b**, only three enzymes completely catalyzed the release of guaiacol in Cabernet Sauvignon, namely, Bglb from *Oscillospiraceae bacterium* (*Ob*BglB), BglB-1 (*Cb*BglB-1), and BglB-2 (*Cb*BglB-2) from *Clostridia bacterium*. These three enzymes also demonstrated shared activity toward **1a**, indicating a potential functional overlap in their ability to catalyze the release of volatile phenols. All three enzymes were from the *Clostridia* bacteria class in ruminant gastrointestinal microbiome and share about 70% sequence identity with each other. To the best of our knowledge, this is the first instance where these three enzymes have been characterized against smoke-associated phenolic glycosides.

### 3.2 Characterization of Promising Candidate *Cb*BglB-1

To accurately characterize the enzyme candidates, it is essential first to validate the HS-SPME GC–MS method, ensuring its reliability and precision in our analysis. It was done by spiking VPs into four matrices: nonsmoke-affected wine and grape samples in both enzymatic and acidified conditions. The basic chemical compositions of the wine and grape samples are shown in Tables S3 and S4. All VPs with the sole exception of 4-ethylguaiaol **3** were introduced at a concentration of 20 μg/L in wine samples and 20 μg/kg in grape samples. For **3**, the concentration was established at 2 μg/L in wine and 2 μg/kg in the berry homogenate. To prepare the four matrices, two approaches were employed: the first involved adding cocktail enzymes to both the wine and berry homogenates, and the second involved acidifying the wine and berry homogenates using HCl to lower the pH to 1.0, without enzyme addition. The spike-recovery results showed that most VPs had good recovery rates in all four matrices, exceeding 80% across the four matrices. For VPs **3**, **9**, and **10**, the recoveries in berry homogenate with enzyme addition were slightly lower at 74, 75, and 80%, respectively, but these values are still within an acceptable range for analytical robustness (Table S5). It is noted that acidified samples yielded generally similar recovery to samples with enzymes. The coefficient of variation (CV) value was used to assess the method’s precision, and all of them were below 20%. It has been reported that artifact formation was particularly possible with guaiacol when using the liquid–liquid extraction method.^13,18^ On the contrary, the HS-SPME method could avoid artifact formation.^13^ For guaiacol **1**, the recovery was optimal, ranging between 96 and 112%, and the coefficient of variation (CV) was less than 10% for all tested matrices, suggesting minimal artifact formation for guaiacol. The results indicated that the current method could give quantitative recovery with good precision and no bias between hydrolysis methods were observed when analyzing the free VPs.

**Figure 3:**
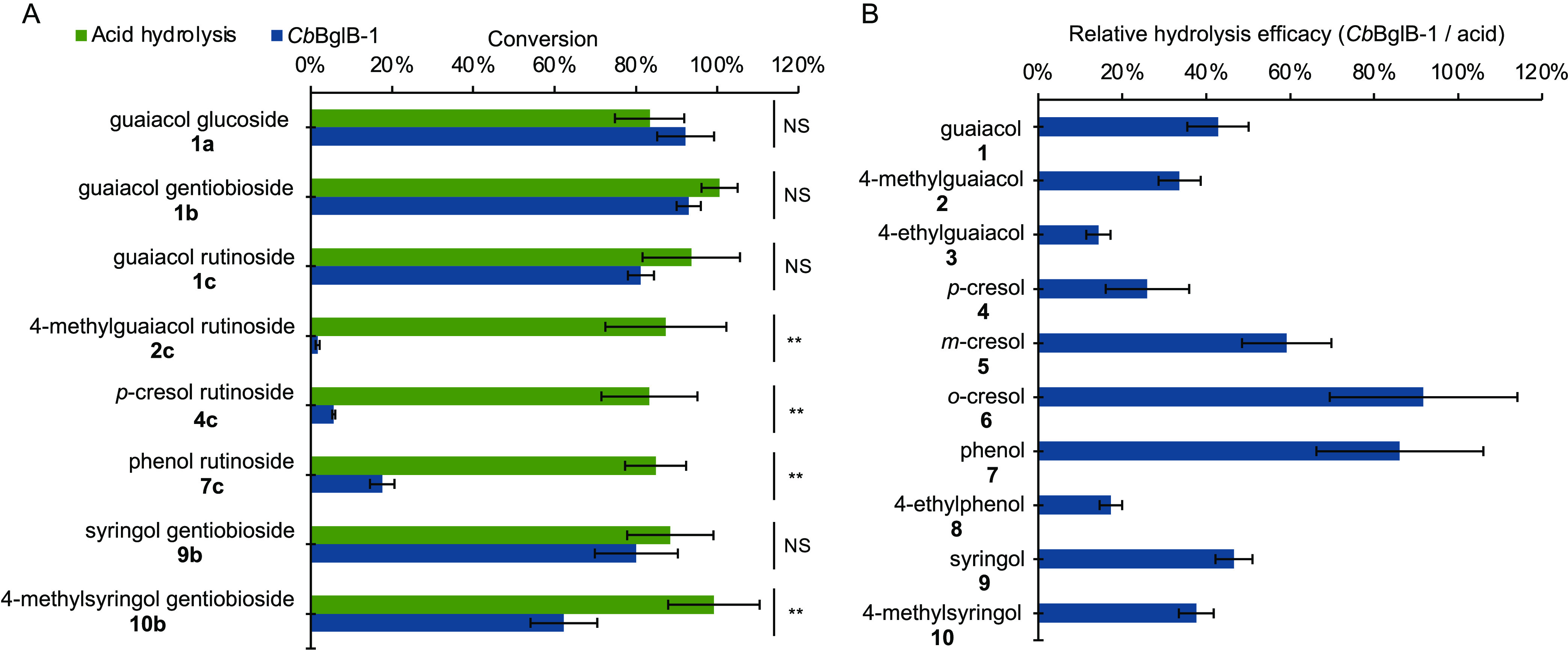
Characterization of *Cb*BglB-1. (A) Fortification experiment was performed to evaluate the ability of *Cb*BglB to convert phenolic glycosides. 40 μg/L of phenolic glycosides were fortified into the baseline wine. The conversion value was calculated by subtracting the final concentration of each VP in baseline wine from those after enzymatic hydrolysis, then dividing by the theoretical yield of each VP calculated based on the mass balance to corresponding VP glycosides. (B) Relative efficacy of *Cb*BglB-1 catalyzed hydrolysis compared to acid hydrolysis in smoke-impacted Cabernet Sauvignon wine. The ratio for each VP was calculated by dividing the total VP release measured after enzymatic hydrolysis by that of acid hydrolysis. Triplicate data were collected. NS denotes not significant (*p*-value >0.05), while ** denotes *p-*value <0.01.

To elect the best candidate among the three outstanding enzymes in the initial screening, we further compared their promiscuous actives and substrate scopes with those of fortification experiments. Eight commercially available β-d-glycosides (guaiacol glucoside **1a**, guaiacol gentiobioside **1b**, guaiacol rutinoside **1c**, 4-methylguaiacol rutinoside **2c**, *p*-cresol rutinoside **4c**, phenol rutinoside **7c**, syringol gentiobioside **9b**, 4-methylsyringol gentiobioside **10b**) (Figure 1A) with diverse VP aglycons and sugar moieties were fortified in nonsmoke-impacted baseline Cabernet Sauvignon with a more realistic concentration of 40 μg/L. The conversion is calculated by subtracting the concentration of VPs recovered from baseline wine (containing naturally occurring VP glycosides) after enzymatic hydrolysis from the concentration of VPs in baseline wine with fortified VP glycosides after enzymatic hydrolysis, as quantified by GC–MS. Similar substrate scope and activity profiles were observed for *Ob*BglB and *Cb*BglB-2. All three enzymes could utilize more than 80% of guaiacol glycosides (**1a, 1b**, and **1c)** as expected and about 80% of **9b** (Figure S4A). All three enzymes displayed a strong preference for gentiobioside **b**. While *Ob*BglB and *Cb*BglB-2 resulted in higher **10b** conversion, *Cb*BglB-1 could utilize **7c** exclusively (Figure 3A). Meanwhile, *Cb*BglB-1 showed a remarkedly higher expression level than *Ob*BglB and *Cb*BglB-2, which was potentially beneficial for industrial applications (Figure S4B). Therefore, we focused on *Cb*BglB-1 as a protein of interest for subsequent testing and optimization.

Using the levels of VPs generated by acid hydrolysis as a benchmark, we could calculate the ratio of each glycoside converted by enzymatic hydrolysis relative to that by acid hydrolysis (Figure 3A and Table S6). Thus, it is essential to conduct validation of the acid hydrolysis process. We performed acid hydrolysis experiments with fortified guaiacol glucoside **1a**, guaiacol gentiobioside **1b**, guaiacol rutinoside **1c**, 4-methylguaiacol rutinoside **2c**, p-cresol rutinoside **4c**, phenol rutinoside **7c**, syringol gentiobioside **9b**, and 4-methylsyringol gentiobioside **10b** at 40 μg/L in wine samples. Conversion/recovery was calculated as detailed in Section 2, which is based on the quantification of free VPs using GC–MS. This calculation involves a mass balance analysis between the free VPs and their bound glycosides (Table S6). The level of conversion achieved by acid hydrolysis for compounds **1b**, **1c**, **2c**, **9b**, and **10b** was notably high, reaching or surpassing 90%. Compounds **1a**, **4c**, and **7c** exhibited relatively lower conversion levels, yet each still maintained a conversion above 80%. In contrast, the enzyme *Cb*BglB-1 demonstrated poor activity characterized by significantly reduced conversion levels for compounds **2c**, **4c**, **7c**, and **10b**. While *Cb*BglB-1 was efficacious at releasing glucosides from VP glucosides **a** and gentiobiosides from VP gentiobiosides **b**, it had a low efficacy in releasing rutinosides for most VP glycosides (Figure 3A). *Cb*BglB-1’s activity levels were also found to be sensitive to the type of aglycon present. This was illustrated by the enzyme’s high activity on compound **1c**, contrasted with its significantly lower activity on compounds **2c**, **4c**, and **7c**, despite the tested compounds (**1c**, **2c**, **4c**, and **7c**) sharing the same rutinoside motif. These findings indicated that while enzymatic hydrolysis via *Cb*BglB-1 was less effective for these VP glycosides in wine, acid hydrolysis exhibited notable catalytic efficacy. Therefore, additional genome mining efforts would be required to find an enzyme capable of efficiently releasing VPs from those bound to a rutinoside sugar motif across a variety of aglycon structures.

A direct comparison was also performed between acid hydrolysis and *Cb*BglB-1 mediated enzymatic hydrolysis of VP glycosides in high smoke-impacted Cabernet Sauvignon wine (Figure 3B and Table S7). Enzymatic hydrolysis achieved less than 90% conversion for the majority of the measured VPs compared to acid hydrolysis, with the majority of VPs between 20 and 50% of the conversion yields observed in acid hydrolysis in high smoke-impacted wine. Although similar efficacy on guaiacol glycosides **1a**, **1b**, and **1c** was observed in the fortified samples (Figure 3A), a lower efficacy of *Cb*Bg1B-1 compared to acid hydrolysis was noted in real-world samples (Figure 3B). This discrepancy may be attributed to the presence of other guaiacol glycosides as well as potential substrate and product inhibition.

### 3.3 Identification of Active Rutinosidases on Volatile Phenol Rutinosides through Genome Mining

The 6-*O*-α-L-rhamnopyranosyl-β-d-glucosidases (rutinosidases; EC 3.2.1.168) belong to the GH5 subfamily and specifically act on the flavonoid diglycosides, including compounds like quercetin 3-*O*-rutinoside, hesperetin 7-*O*-rutinoside, kaempferol-3-*O*-rutinoside, and naringenin 7-*O*-neohesperidoside.^31^ Notable rutinosidases have been reported from several species including *Acremonium sp*. DSM 24697, *Actinoplanes missouriensis*, *Aspergillus niger* K2, and *Aspergillus oryzae* RIB40. Advancements have been made recently in understanding the properties of these enzymes and the crystal structures of rutinosidase from *A. niger* K2 (*Ani*Rut),^32^ and rutinosidase from *A. oryzae* RIB40 (*Aory*Rut)^33^ were deciphered to shed light on the substrate specificity. Remarkedly, *Aory*Rut is capable of accommodating various flavonoids including both 7-*O*-linked and 3-*O*-linked flavonoids, possibly contributed by the flexible loop located at the substrate entrance. While there is considerable interest in its application within the food industry, the exploration of the enzymes’ substrate scope beyond flavonoid glycosides remains limited. We performed genome mining in a nonexhaustive manner with a particular emphasis on identifying rutinosidase activity against 4-methylguaiacol rutinoside **2c** among the collection of selected proteins.

**Figure 4:**
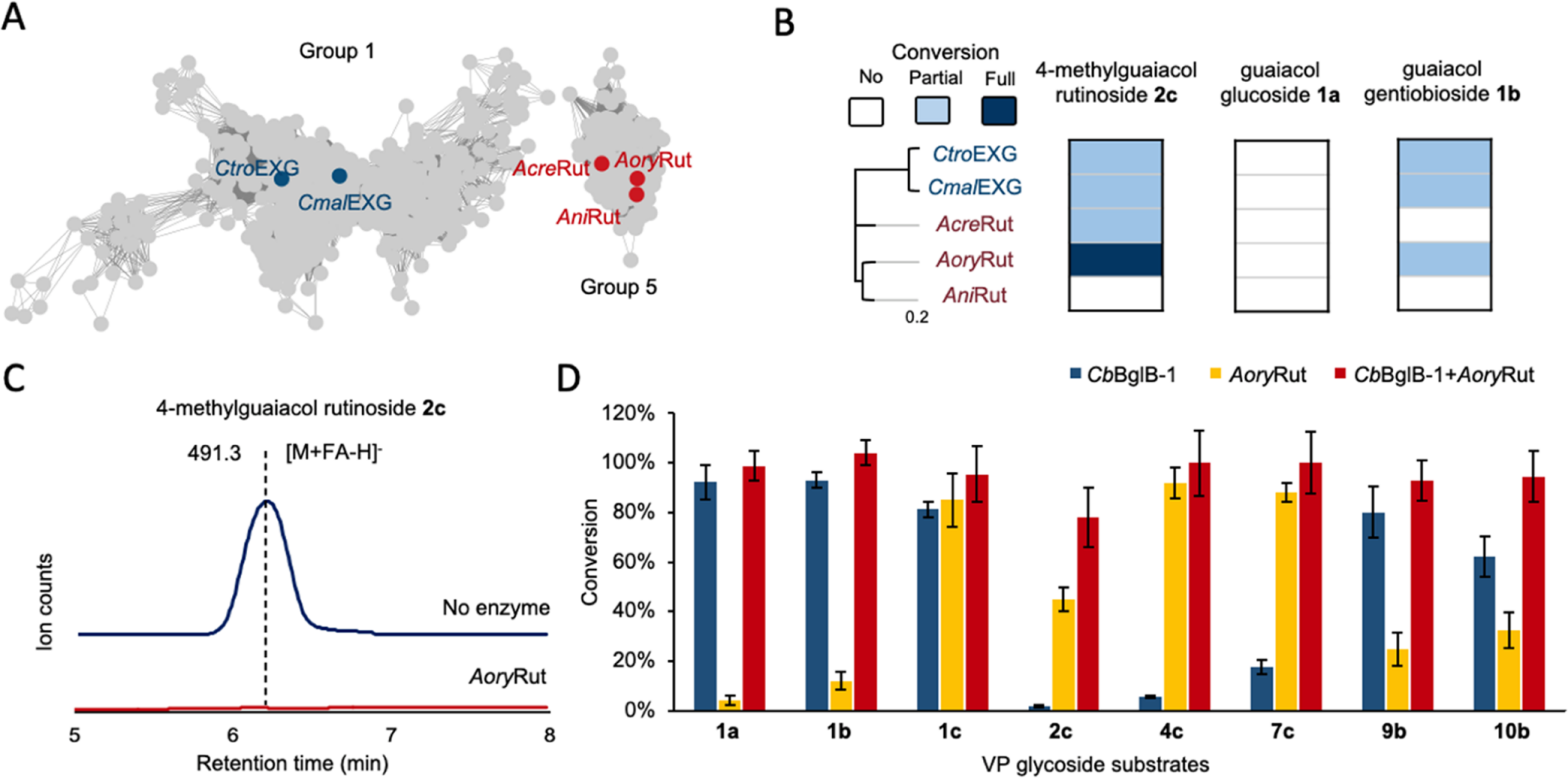
Identification of active rutinosidases on smoke-related VP rutinosides. (A) SSN of the GH5 enzyme family was constructed and only the groups where the tested enzyme sequences were located were shown. (B) Semiquantitative heatmap of the degrees of conversion by rutinosidase candidates on **2c**, **1a**, and **1b** in wine. Candidates were mixed with baseline wine which had been fortified with 4.5 mg/mL of substrate. The reaction was at 37 °C for 4 h duration. (C) Utilization of LC–MS analysis for rutinosidase screening in wine. As an example, AoryRut could completely degrade **2c** indicated by the disappearance of the corresponding peak in MS traces. (D) Fortification experiment involving AoryRut and enzyme cocktail of CbGglB-1 and AoryRut against various glycosides fortified into a baseline wine. Triplicate data were collected.

GH5 SSN composed of about 67,000 genes was built and previously identified rutinosidases such as *Aory*Rut and *Ani*Rut centered on group 5 (Figure S5). We assigned a higher preference to enzymes situated in groups 1 and 5 to ensure that the chosen representatives spanned across a wide sequence space, while also leveraging the accessible knowledge base (Figure 4A). The genes encoding *Ctro*EXG, *Cmal*EXG, *Acre*Rut, *Aory*Rut, and *Ani*Rut with average sequence identity around 50% were selected, and their corresponding proteins expressed in *E. coli* were purified and their semiquantitative performance on **2c** was evaluated by LC–MS. While 4 out of 5 showed activity, *Aory*Rut was the sole enzyme that could utilize **2c** (Figure 4B,C). We also examined their ability to utilize **1a** and **1b**, and the result showed that 3 out of 5 were active toward **1b** but none of them were active on **1a** (Figure 4B). The result was consistent with a previous report that *Aory*Rut demonstrated different substrate promiscuities to *Ani*Rut and the specificity is determined by both glycone types in flavonoid glycosides and the aglycone moiety and generally prefers disaccharide glycosides to monosaccharide glycosides.^33^

While *Aory*Rut appeared to fully convert substrate **2c** in the LC–MS trace (Figure 4C), its inability to completely convert **2c** detected by GC–MS suggested a discrepancy (Figure 4D). This might be due to the LC–MS’s lower sensitivity limit, set at the mg/L level, which could overlook intact substrates below the limit of detection. The decision to use mg/L level substrates was to ensure robust enzyme-catalyzed reaction rates, essential for identifying active enzymes. The partial conversion observed at the lower μg/L concentrations could be a result of GC–MS’s higher sensitivity and lower activity levels with decreased substrate concentrations.

*Cb*BglB-1 is annotated as a GH1 enzyme family in which the enzymes typically exhibit exacting activity with the progressive release of monosaccharides from these linkages.^34^*Aory*Rut has been classified as a GH5 diglycosidase and can cleave the entire disaccharidic moiety from the aglycone.^35^ By strategically combining enzymes of *Cb*BglB-1 and *Aory*Rut with varied action modes, it became possible to target a broader range of glycosidic bonds and is likely to yield diversified glycosidic bond cleavage in smoke-derived VP glycosides (Figure 4D and Table S6). The combination achieved more than 90% conversion on nearly all tested glycosides, except for **2c**, which had around 75% conversion. It was postulated that the enzyme cocktail could serve as a promising candidate for comparison against the conventional acid hydrolysis approach.

### 3.4 Hydrolysis Efficacy Comparison between Enzymatic Hydrolysis and Acid Hydrolysis

It was essential to establish the optimal parameters that directly affect the process of enzymatic hydrolysis before we deployed the enzyme cocktail in the samples to compare with acid hydrolysis. Thus, we examined two important parameters in order: incubation time and enzyme loading (Figure S7). To fine-tune the incubation time, we tested various reaction durations including 0.25, 1, 4, and 24 h. The time-course experiment indicated that the reaction achieved equilibrium in 4 h and the extension of reaction time would not necessarily yield more VPs (Figure S7A). Therefore, the enzyme reactions could be completely stopped by adding 40% w/v NaCl after 4 h (Figure S8). To determine the best enzyme loading value, we mixed the high smoke-impacted Cabernet Sauvignon wine with varying ratios and concentrations of constituent enzymes in the cocktail. We first assessed *Cb*BglB with five different loading amounts, resulting in five varying final enzyme concentrations of *Cb*BglB-1 (0.4, 0.8, 2, 4, and 5 mg/mL), and compared the outcomes of total VPs. While the higher concentration of *Cb*BglB-1 up to 4 mg/mL resulted in an increasing summed amount of VPs, there was no significant difference when comparing the results using 4 and 5 mg/mL enzyme (Figure S7B). Thus, 4 mg/mL of *Cb*BglB-1 was applied in follow-up experiments with the assumption that loading more than 4 mg/mL of *Cb*BglB-1 would not generate more VPs in the matrix of present smoke-tainted wine. Next, various concentrations of *Aory*Rut were supplemented: 0.2, 0.5, 0.8, 1.0, and 1.2 mg/mL. The quantity of total VPs increased along with the concentration of *Aory*Rut, up to a maximum of 1.0 mg/mL. A higher concentration of *Aory*Rut than 1.0 mg/mL did not make a significant difference in total VP levels (Figure S7C). Overall, the enzyme cocktail performed well when the incubation time was at least 4 h and the concentrations of *Cb*BglB-1 and *Aory*Rut were 4 and 1 mg/mL, respectively.

Previous research has highlighted the existence of commercial enzyme preparations capable of hydrolyzing smoke-derived VP glycosides in wine.^36^ A comparative study of hydrolysis using glycosidase 2 (Rapidase Revelation Aroma), *Cb*BglB-1, and *Aory*Rut was done (Figure S9). While glycosidase 2 increased the concentration of all free VPs, its activity was significantly lower than that of *Cb*BglB-1, with the total VP concentration reaching only about 65% of that produced by *Cb*BglB-1-catalyzed reactions. The final accumulated concentration of VPs catalyzed by glycosidase 2 was approximately 40% of that achieved by a cocktail of *Cb*BglB-1 and *Aory*Rut (Figure S9). Thus, glycosidase 2 exhibited suboptimal activity for VP glycoside quantification and might not be directly used for this purpose without additional optimization.

To further corroborate the efficacy of the enzyme cocktail, we implemented a direct quantification strategy for VP glycosides in wine and berries. We mixed nonsmoke-affected samples with known VP glycoside substrates and then conducted LC–MS/MS analysis both before and after subjecting them to enzymatic and acid hydrolysis. This method allowed us to measure the conversion of VP glycosides accurately, providing clear evidence of the effectiveness of the enzyme cocktail in processing these compounds.^6,37^ The results confirmed that both acidic and enzymatic hydrolysis successfully converted all VP glycosides (Table 1). In wine, enzymatic hydrolysis showed slightly enhanced effectiveness over acid hydrolysis for substrates **1a**, **1b**, **2c**, **4c**, and **7c**, though it was less efficient for **1c**, **8b**, and **10b**. Enzymatic hydrolysis achieved a minimum conversion rate of 88% in wine for all VP glycosides. For grape samples, enzymatic hydrolysis generally yielded higher conversion rates for almost all VP glycosides with **10b** being the sole exception. The direct measurement of the depletion of VP glycosides was consistent with the formation of free VPs, thus reinforcing the validity of our approach.

**Table 1:** Quantification Results of VP Glycosides through LC–MS/MS in the Spike-Recovery Experiment^a^

sample	**1a** (μg/L)	**1b** (μg/L)	**1c** (μg/L)	**2c** (μg/L)	**4c** (μg/L)	**7c** (μg/L)	**9b** (μg/L)	**10b** (μg/L)
wine with spiked glycosides (*n* = 1)	37.67	32.41	41.10	43.02	34.59	39.73	40.46	36.00
wine with spiked glycosides after acid hydrolysis (*n* = 3)	0.36 ± 0.06	0.42 ± 0.05	0.73 ± 0.09	0.36 ± 0.07	1.08 ± 0.15	1.82 ± 0.15	0.31 ± 0.04	0.17 ± 0.03
wine with spiked glycosides after enzymatic hydrolysis (*n* = 3)	ND	ND	4.91 ± 0.42	ND	1.01 ± 0.03	0.28 ± 0.13	0.48 ± 0.15	4.09 ± 0.38
berry with spiked glycosides (*n* = 1)	38.03	31.56	39.78	33.66	24.75	38.21	41.32	43.95
berry with spiked glycosides after acid hydrolysis (*n* = 3)	0.78 ± 0.22	0.49 ± 0.11	0.62 ± 0.08	0.36 ± 0.07	0.93 ± 0.21	1.34 ± 0.45	0.56 ± 0.15	0.4 ± 0.11
berry with spiked glycosides after enzymatic hydrolysis (*n* = 3)	ND	ND	ND	ND	0.63 ± 0.03	ND	ND	0.58 ± 0.07

a ND = not detectable in all samples. The limit of quantification of all compounds is <0.1 ng/L based on ten times the signal-to-noise ratio.

**Figure 5:**
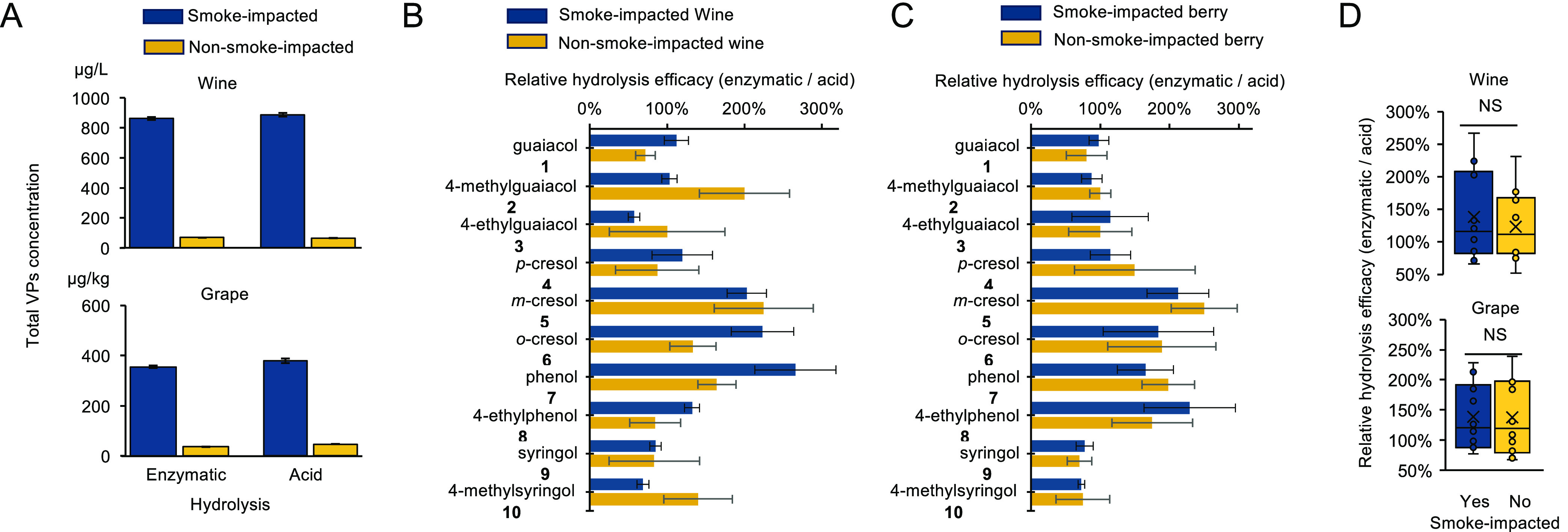
Application of enzyme cocktail to Cabernet Sauvignon wine and Cabernet Sauvignon grape with different levels of smoke impact. (A) Both acid hydrolysis and enzymatic hydrolysis demonstrated significantly higher total VP concentrations with the sum of all 10 VPs in smoke-impacted wine and grape than those in nonsmoke-impacted samples. (B) Relative efficacy of enzymatic hydrolysis to acid hydrolysis for each bound VP in wine. (C) Relative efficacy of enzymatic hydrolysis to acid hydrolysis for each bound VP in grape berries. (D) Box and whisker plots of the relative efficacy of enzymatic hydrolysis to acid hydrolysis for glycosides of each VP (median (line), mean (X)). The enzymatic cocktail consistently achieved comparable efficacy of acid hydrolysis regardless of the sample types. NS denotes not significant (*p*-value >0.05). Experiments were conducted in triplicate.

To assess the compatibility, we conducted enzymatic hydrolysis with the enzyme cocktail on Cabernet Sauvignon wines and grape berries, categorized into smoke-impacted and nonsmoke-impacted groups. Reflected by the total concentration of VPs, both wine and grape samples impacted by smoke contained significantly elevated concentrations of VP glycosides compared to those samples unaffected by smoke, and the results validated the potential of the hydrolysis method for binary and qualitative assessments of smoke impact (Figure 5A and Table S7). Among the VP glycosides, glycosides of syringol **9** calculated from the subtraction of free 51.17 μg/L from total (after enzymatic hydrolysis) 407.7 μg/L were the most abundant in smoked-impacted Cabernet Sauvignon with a concentration of 356.5 μg/L (Table S7). Compound **9b** was one of the predominant glycosides in high smoke-tainted Cabernet Sauvignon, and our result is in accordance with prior studies.^11^ The concentrations of **3** and **8** in smoke-impacted wine after enzymatic hydrolysis were approximately 10-fold higher than those in the baseline, which showed that **3** and **8**, which are normally associated with *Brettanomyces* yeast growth,^38^ can also be present as a consequence of smoke exposure (Table S7). Compounds **1** and **2** which are typically regarded as markers of smoke taint exhibited a significant increase following enzymatic hydrolysis, and their concentrations were clearly distinguishable between smoke-impacted samples and nonsmoke-impacted samples.

We conducted a detailed analysis to compare the differences between enzymatic and acidic hydrolysis in wine samples (Figure 5B). The enzymatic hydrolysis led to a higher conversion of half of the bound VPs in both smoke-impacted and nonsmoke-impacted wines, albeit for different VPs (Figure 5B). Enzymatic hydrolysis significantly outperformed acid hydrolysis for **5**, **6**, and **7** glycosides with the range of 150–300% higher conversion. The enzymatic hydrolysis displayed a comparable effectiveness for compounds **1**, **2**, **4**, **8**, **9**, and **10** glycosides, despite the varying ratios seen in the smoked and unsmoked wines. It is worth mentioning that aligned with the established literature, we found that syringol **9** and 4-methylsyringol **10** were effectively released by acid hydrolysis.^4^

To alleviate the economic consequences of producing smoke-affected wines, it is imperative to determine the quantities of both free and bound VPs in grapes prior to fermentation. We first performed a spike-recovery of VP glycoside experiment in berry homogenates to prove the efficacy of acid hydrolysis and enzymatic hydrolysis. The result showed that both acid hydrolysis and enzymatic hydrolysis achieved a conversion degree exceeding 90% for all VP glycosides (Figure S10 and Table S6). Enzymatic hydrolysis of smoke-impacted Cabernet Sauvignon grapes and control grapes was then studied (Figure 5A,C and Table S7). This allowed us to assess the method’s compatibility with grapes. Following a similar trend as observed in smoke-impacted wine, total VPs in posthydrolysis of smoke-impacted grape berries were considerably higher than those for control grapes (Figure 5A), and compound **9** persisted as the most abundant VP after hydrolysis in smoke-impacted grape berries (Table S7). The existing literature suggests that fermentation by yeast and the aging process can hydrolyze the bound VPs; smoke-exposed berries therefore should theoretically contain a greater proportion of bound VPs before fermentation or aging.^39^ Our findings supported this theory, as we observed a notable increase in the ratio of bound to free VPs in smoke-impacted grapes relative to that in wines.

Consistent with the performance in wine samples, enzymatic hydrolysis of berries showed a 150–300% increase in conversion than acid hydrolysis for bound forms of **5**, **6**, and **7** (Figure 5C). Interestingly, enzymatic hydrolysis substantially excelled for the glycosides of **8** in both types of grape samples, whereas its performance was only marginally superior in smoke-impacted wine samples (Figure 5C). The conversion rates for all other VPs between enzymatic hydrolysis and acid hydrolysis were nearly identical despite a minor increase of enzymatic hydrolysis for bound compounds **3** and **4**. It was noted that the ratios of enzymatic hydrolysis to acid hydrolysis for all phenolic glycosides exhibited less variation in smoke-impacted and nonsmoke-impacted grapes than in wine samples, illustrating the operational stability in grapes.

Finally, relative hydrolysis efficiencies of enzymatic to acid for individual bound VPs were mapped into box and whisker plots to summarize the value distribution across different sample types (Figure 5D). The enzymatic hydrolysis method consistently showed a slightly higher effectiveness in converting bound VPs compared to that of acid hydrolysis. In wine samples, there was an approximate median of 1.2-fold increase, while in grape samples, the mean increase was 1.35-fold. Moreover, the enzymatic hydrolysis method demonstrated near-identical performance regardless of the degree of smoke impact, showcasing the robustness and consistency of the enzymatic hydrolysis approach.

Smoke taint-associated VP glycosides have been profiled in wine and grape berries in previous studies.^6,37^ Alongside the VP glycoside substrates tested in the current work, diglycosides with a terminal pentose were also tentatively identified as abundant glycosidic conjugates. The sum of these diglycosides was reported to exceed 70% of the total guaiacol glycoconjugates in smoke-exposed grapes. These diglycosides, primarily linked to glucose, include specific pentoses such as apiofuranose, arabinofuranose, arabinopyranose, and xylopyranose. Their significant presence also plays an essential role in the development of aroma profiles in grapes and wines. Hydrolysis methods, such as enzymatic hydrolysis, may demonstrate effectiveness on these glycosides owing to their substrate promiscuity and potentially allow for a broader analysis of VP glycosides. Meanwhile, a key focus for future research is isolation or synthesis of these noncommercially available VP glycosides and the characterization of enzymes against them to better understand the substrate scope of the enzyme cocktail.

Utilizing enzymatic hydrolysis has the potential to provide several notable advantages. First, enzymatic hydrolysis and acid hydrolysis are comparable in terms of their effectiveness. Second, acid hydrolysis is well-known to be sensitive to conditions and handling, making it difficult to standardize across laboratories. Conversely, enzymatic hydrolysis operates under milder conditions and avoids the use of harsh chemicals. This provides a safer work environment, which is an important consideration in laboratory settings. Third, the reduced sample preparation, such as pH titration, makes enzymatic hydrolysis an efficient choice for high throughput. This high-throughput capability is particularly beneficial for grape growers and winemakers, allowing for prompt decision-making, especially during fire seasons. Fourth, the method is cost-effective and eliminates the need for high-cost and low-throughput LC–MS/MS-based analytics for commercial laboratories.

In order to develop accurate decision-making tools, large data sets covering a wide variety of grapes, winemaking conditions, environments, and seasons will be needed to fully understand the relationship between VP levels and smoke-taint perception and acceptance.^1,40^ To facilitate development of these data sets, a robust, easy-to-use, inexpensive, and accurate method is required to create analytics around smoke taint. This has been challenging in the industry, to date, given the limited ability to accurately measure bound glycosides and overall low-throughput methodologies that are highly sensitive to sample preparation requirements. We believe that our proposed method can accelerate this process owing to its intrinsic high-throughput and scalable characteristics. As a standardized enzymatic method can be applied to both wines and grape berries with a low-smoke impact, it becomes feasible to compile baseline data as well. We foresee this paving the way to a more profound understanding of the relationship between smoke-impacted levels and various factors like environmental, geographical location, and grape and wine production variables.

## Supporting Information Available

SSN of GH1; preliminary screening of active GH1; representative LC–MS traces for activity analysis on 1a and 1b in buffer; close comparison of three GH1 candidates; SSN of GH5; SDS-PAGE gel of the enzyme cocktail; process optimization of application of the enzyme cocktail in smoke-tainted wine; NaCl addition halted the enzyme reactions; efficacy comparison between enzyme cocktail and commercial enzyme Rapidase; efficacy comparison between enzymatic and acid hydrolysis in grapes; MRM calibration table from commercial standards for individual bound glycoside analysis; quantify, qualify ions and calibration curve details for GC–MS; basic chemical analysis of wine samples; basic chemical analysis of grape samples; recovery and coefficient of variation (CV) of spiked VPs from wine and berry homogenate; mass balance of VP glycosides and free VPs produced in spike-recovery experiments; concentration of free VPs and total VPs after two hydrolysis methods; genes and NCBI/Uniprot identifiers encoding screened enzymes (PDF)
