# Supplementary material for: Discovery of Potent Glycosidases Enables Quantification of Smoke-Derived Phenolic Glycosides through Enzymatic Hydrolysis
Source: J Agric Food Chem. 2024 May 10;72(20):11617–28. doi: 10.1021/acs.jafc.4c01247 (PMC11117406; doi:10.1021/acs.jafc.4c01247)
Supplement: Supplementary file 1 — jf4c01247_si_001.pdf [file jf4c01247_si_001.pdf]

Supporting Information

The discovery of potent glycosidases enables quantification of smoke-derived phenolic glycosides through enzymatic hydrolysis

Youtian Cui[a,h], Mary Riley[a,b], Marcus V. Moreno[a], Mateo M. Cepeda[c], Ignacio Arias Perez[d], Yan Wen[d], Lik Xian Lim[e,f], Eric Andre[a], An Nguyen[a], Cody Liu[a], Larry Lerno[d,g], Patrick K Nichols[h], Harold Schmitz[i,j,k], Ilias Tagkopoulos[a,l,m], James A. Kennedy[h], Anita Oberholster[d*], Justin B. Siegel[a,b,c,n*]

[a] Genome Center, University of California, Davis, 95616, United States of America, [b] Microbiology Graduate Group, University of California, Davis, 95616, United States of America, [c] Department of Chemistry, University of California, Davis, 95616, United States of America, [d] Department of Viticulture & Enology, University of California, Davis, 95616, United States of America, [e] Department of Food Science & Technology, University of California, Davis, 95616, United States of America, [f] UC Davis Coffee Center, University of California, Davis, 95616, United States of America, [g] Food Safety and Measurement Facility, University of California, Davis, 95616, United States of America, [h] VinZyme, LLC, Davis, 95616, United States of America, [i] March Capital US, LLC, Davis, 95616, United States of America, [j] T.O.P., LLC, Davis, 95616, United States of America, [k] Graduate School of Management, University of California, Davis, 95616, United States of America, [l] Department of Computer Science, USDA/NSF AI Institute for Next Generation Food Systems (AIFS), University of California, Davis, 95616, United States of America, [m] PIPA, LLC, Davis, 95616, United States of America, [n] Department of Biochemistry and Molecular Medicine, University of California, Davis, 95616, United States of America

Supporting Information Table of Contents

1. Figure S1 – SSN of GH1
2. Figure S2 – Preliminary screening of active GH1
3. Figure S3 – Representative LC-MS traces for activity analysis on **1a** and **1b** in buffer.
4. Figure S4 – Close comparison of three GH1 candidates.
5. Figure S5 – SSN of GH5.
6. Figure S6 – SDS-PAGE gel of the enzyme cocktail.
7. Figure S7 – Process optimization of application of the enzyme cocktail in smoke-tainted wine.
8. Figure S8 – NaCl addition halted the enzyme reactions.
9. Figure S9 – Efficacy comparison between enzyme cocktail and commercial enzyme rapidase
10. Figure S10 – Efficacy comparison between enzymatic and acid hydrolysis in grapes
11. Table S1 – MRM calibration table from commercial standards for individual bound glycoside analysis
12. Table S2 – Quantify, qualify ions and calibration curve details for GC-MS
13. Table S3 – Basic chemical analysis of wine samples
14. Table S4 –.Basic chemical analysis of grape samples
15. Table S5 – Recovery and coefficient of variation (CV) (n=3) of spiked VPs from wine and berry homogenate.
16. Table S6 – Mass balance of VP glycosides and free VPs produced in spike-recovery experiments
16. Table S7 – Concentration of free VPs and total VPs after two hydrolysis methods
17. Table S8 – Genes and NCBI/Uniprot identifiers encoding screened enzymes

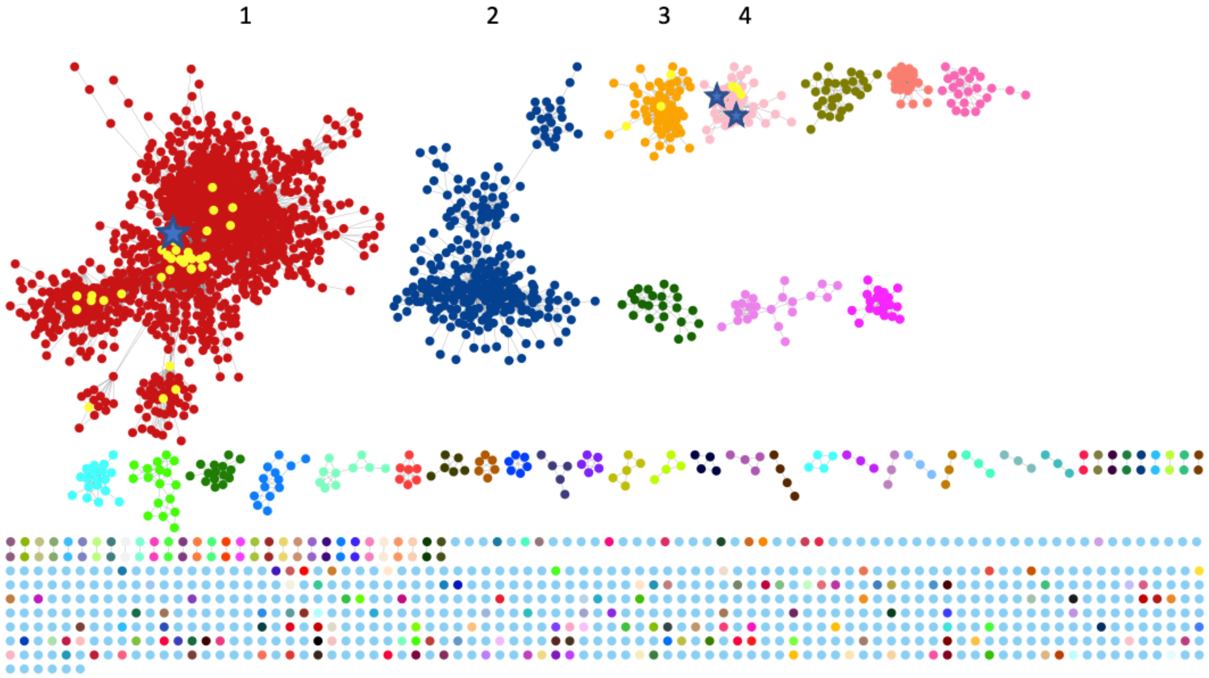

Figure S1. Sequence similarity network of GH1 family. The nodes containing screened enzyme candidates were highlighted in bright yellow.

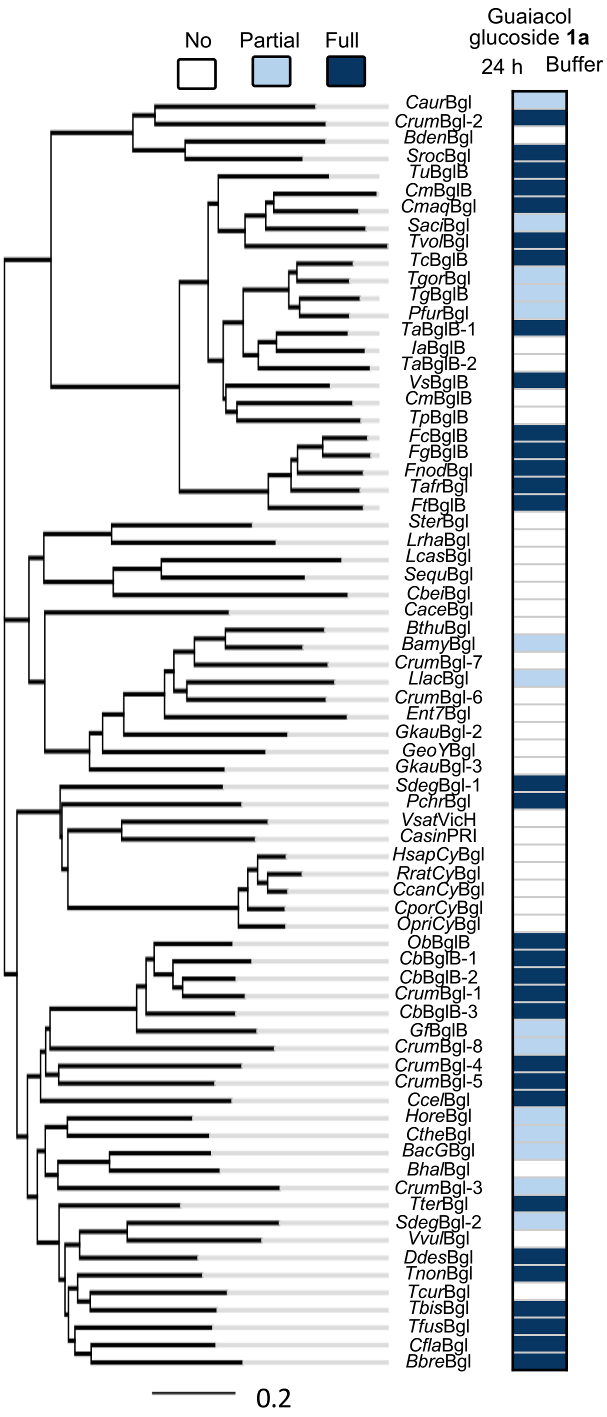

Figure S2. Preliminary screening of active GH1 on compound **1a** at 4.5 mg/L in acetic buffer pH 3.5 for 24 hours' reaction.

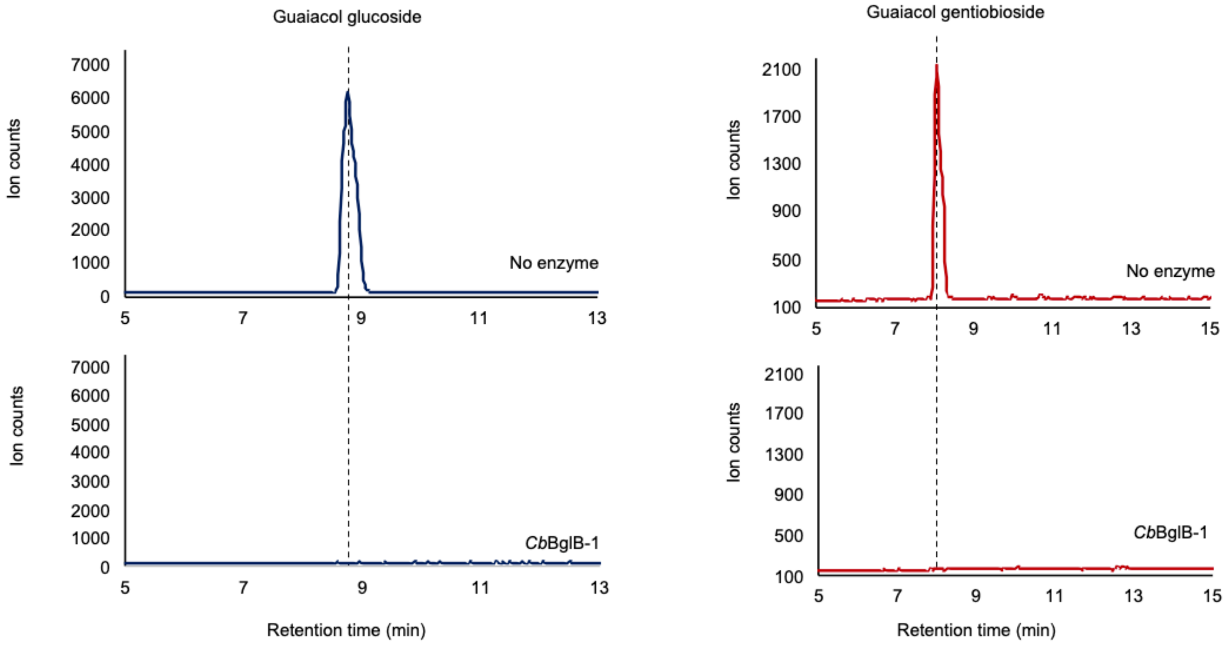

Figure S3. Representative LC-MS traces for activity analysis on **1a** and **1b** in buffer.

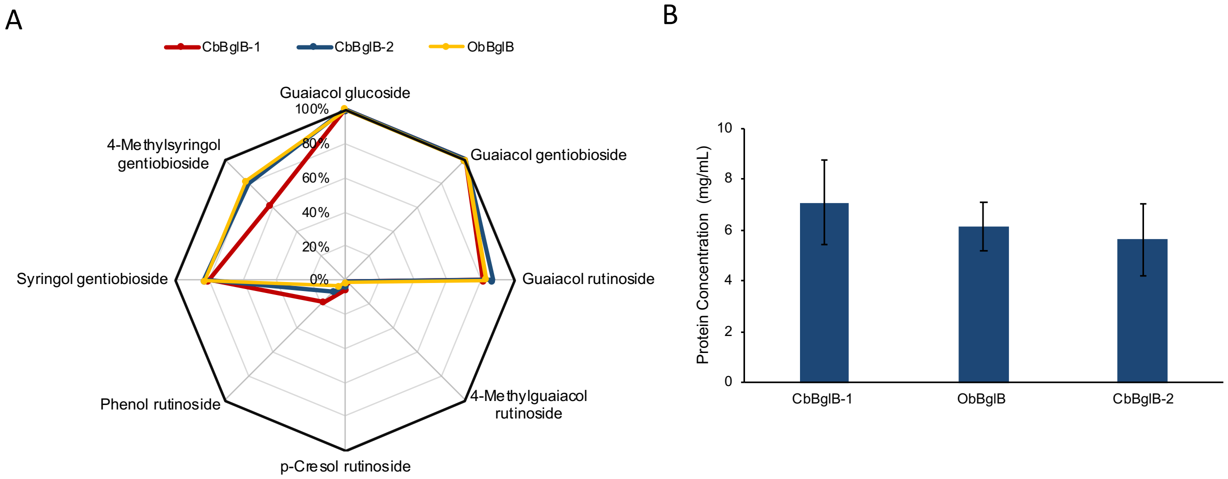

Figure S4. Close comparison of three GH1 candidates that show activities on **1a** and **1b** in buffer and wine. (A)The activity profiles of three candidates. *Cb*BglB-1 was the only candidate capable of using phenol rutinoside.(B) The protein concentrations of the candidates. All proteins were expressed in *E.coli* in 500 mL Terrific Broth culture, purified through cobalt IMAC and quantified through A_{280}.

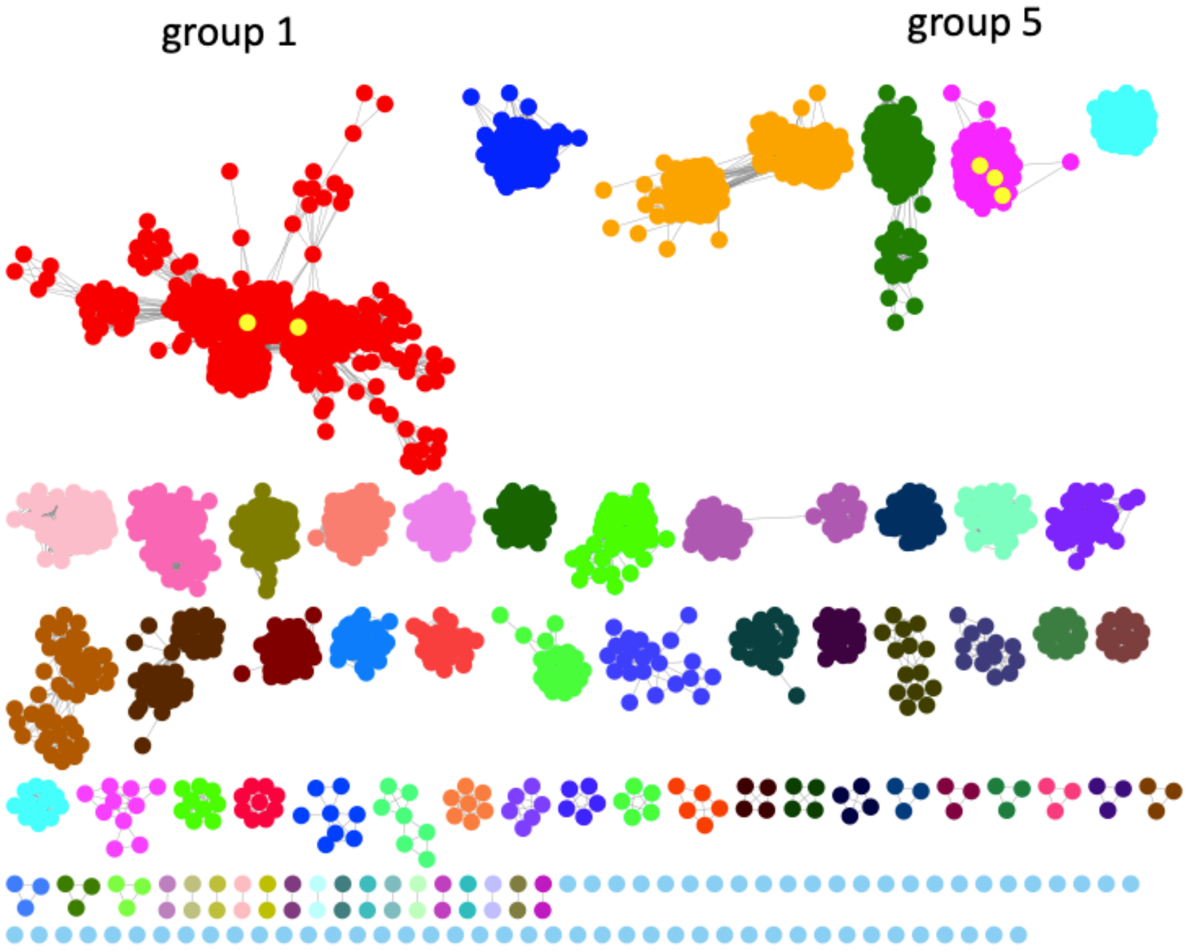

Figure S5. SSN of GH5. The nodes containing screened enzyme candidates were highlighted in bright yellow and were focused in group 1 and group 5.

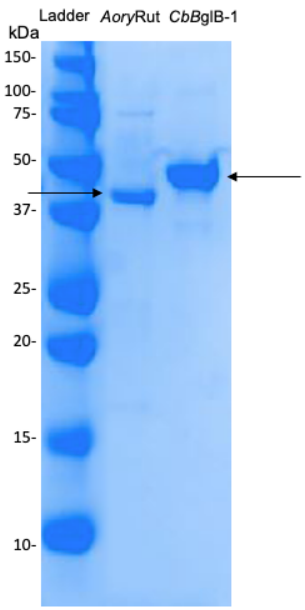

Figure S6. SDS-PAGE gel of the enzyme cocktail. *Aory*Rut has a molecular weight of 41.850 kDa and *Cb*BglB-1 has a molecular weight of 50.964 kDa

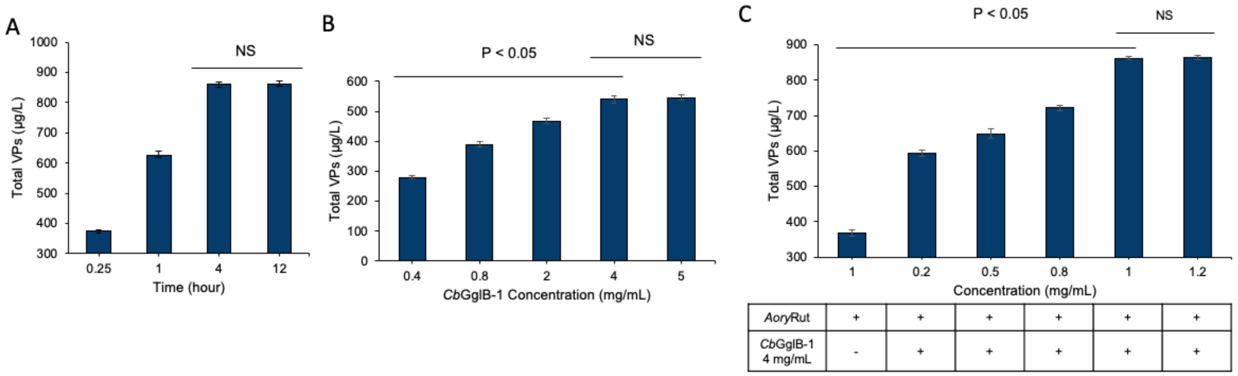

Figure S7. Process optimization of application of the enzyme cocktail in smoke-tainted wine. The enzyme loading amount and reaction duration were optimized. (A) Optimization of reaction duration. (B) Optimization of loading concentration of *Cb*GglB-1in smoke-impacted wine. (C) Optimization of loading concentration of *Acry*Rut in smoke-impacted wine. P<0.05 denotes significant difference; NS denotes not significant.

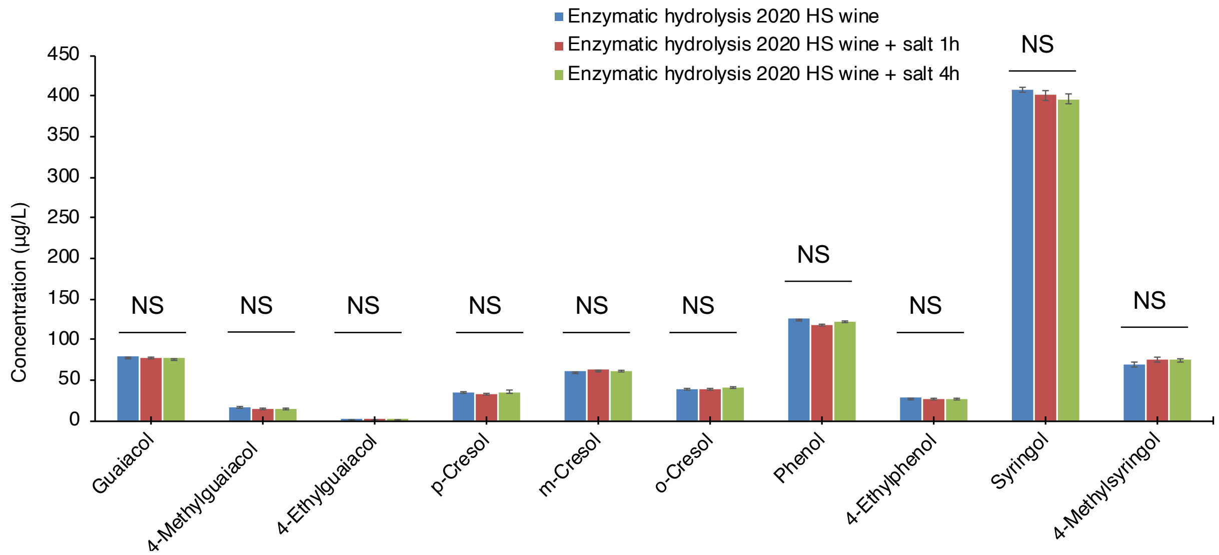

Figure S8. Addition of 40% w/v NaCl halted the enzyme reactions. NS denotes not significant (*p*-value > 0.05)

A

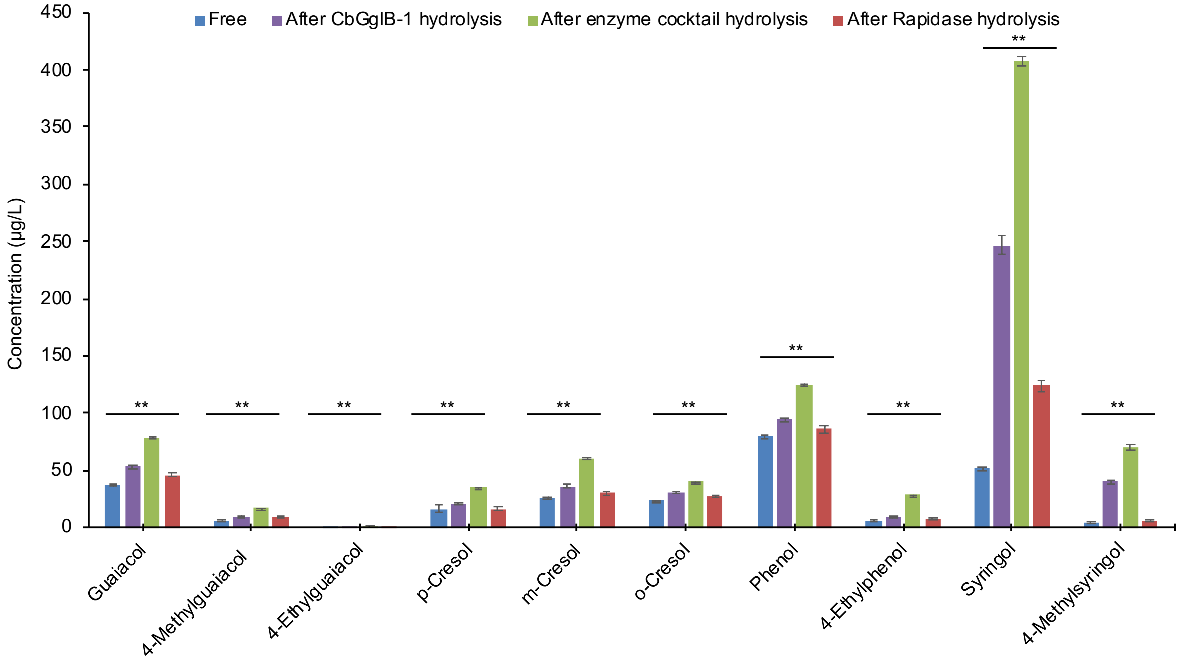

B

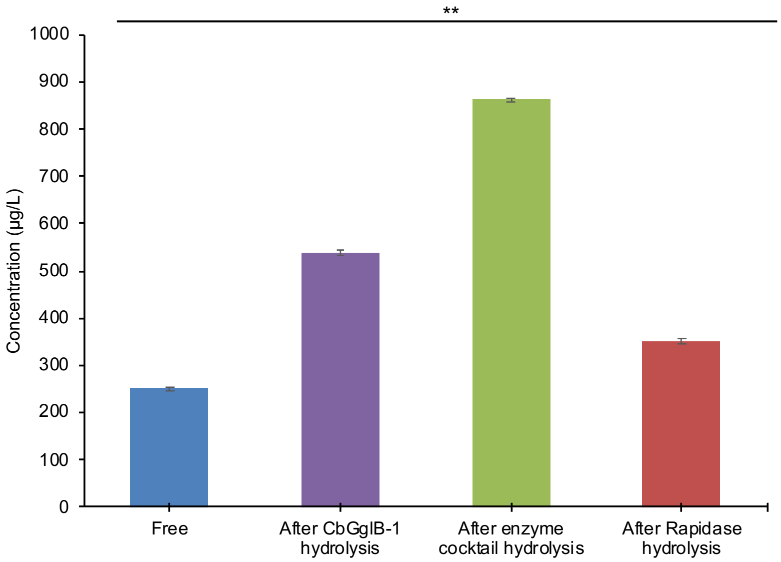

Figure S9. Efficacy comparison between enzyme cocktail and commercial glycosidase 2 (rapidase). (A) Individual VP concentration before (Free) and after enzymatic hydrolysis of high smoke-impacted wine. (B)The sum of VPs concentration before (Free) and after enzymatic hydrolysis of high smoke-impacted wine. Biological triplicates were performed. Rapidase = DSM Rapidase Revelation Aroma with final concentration of 0.03g/L in samples. ** denotes statistically significant with p-value < 0.05

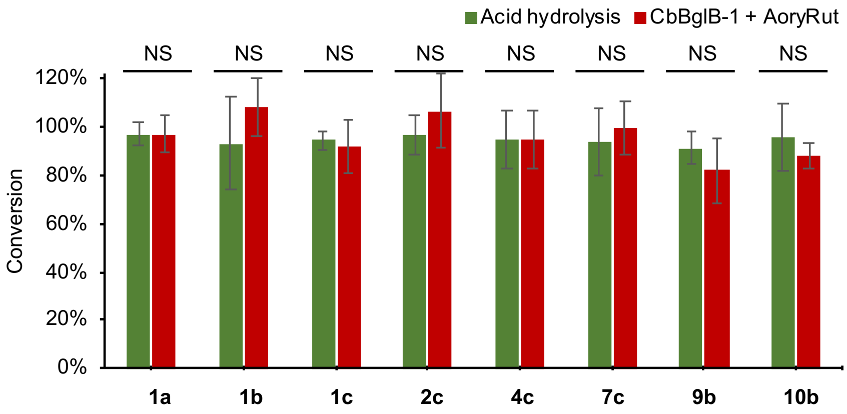

Figure S10. Efficacy comparison between enzymatic and acid hydrolysis in grapes. N=3. NS denotes not significant with *p*-value > 0.05

Table S1: MRM calibration table from commercial standards for individual bound glycoside analysis

Compound Name	Precursor Ion	Product Ion	Fragmentor (V)	Collision Energy (V)	Retention Time (min)	Retention Window	Polarity
Guaiacol gentiobioside	466	325	90	5	3.05	0.8	Positive
Guaiacol gentiobioside	466	163	90	10	3.05	0.8	Positive
Guaiacol gentiobioside	466	145	90	20	3.05	0.8	Positive
d3-Guaiacol gentiobioside	469	325	90	5	3.05	0.8	Positive
d3-Guaiacol gentiobioside	469	163	90	10	3.05	0.8	Positive
d3-Syringol gentiobioside	501	163	90	15	3.45	0.8	Positive
d3-Syringol gentiobioside	501	145	90	20	3.45	0.8	Positive
Syringol gentiobioside	496	325	90	5	3.5	0.8	Positive
Syringol gentiobioside	496	163	90	15	3.5	0.8	Positive
Syringol gentiobioside	496	145	90	20	3.5	0.8	Positive
Guaiacol glucoside	304	163	70	5	3.66	0.8	Positive
Guaiacol glucoside	304	145	70	5	3.66	0.8	Positive
Guaiacol glucoside	304	125	70	5	3.66	0.8	Positive
d5-Phenol rutinoside	425	309	90	5	3.69	0.8	Positive
d5-Phenol rutinoside	425	147	90	10	3.69	0.8	Positive
Phenol rutinoside	420	309	90	5	3.76	0.8	Positive
Phenol rutinoside	420	164	90	5	3.76	0.8	Positive
Phenol rutinoside	420	147	90	10	3.76	0.8	Positive
d3-Guaiacol rutinoside	453	309	90	5	4.54	0.8	Positive
d3-Guaiacol rutinoside	453	147	90	10	4.54	0.8	Positive
Guaiacol rutinoside	450	309	90	5	4.58	0.8	Positive
Guaiacol rutinoside	450	164	90	10	4.58	0.8	Positive
Guaiacol rutinoside	450	147	90	10	4.58	0.8	Positive
4-Methylsyringol gentiobioside	510	325	90	10	5.08	0.8	Positive
4-Methylsyringol gentiobioside	510	163	90	15	5.08	0.8	Positive
4-Methylsyringol gentiobioside	510	145	90	20	5.08	0.8	Positive
d5-Cresol rutinoside	439	309	90	5	5.56	0.8	Positive
d5-Cresol rutinoside	439	147	90	10	5.56	0.8	Positive
Cresol rutinoside	434	309	90	5	5.67	0.8	Positive
Cresol rutinoside	434	164	90	5	5.67	0.8	Positive
Cresol rutinoside	434	147	90	10	5.67	0.8	Positive
d3-4-Methylguaiacol rutinoside	467	309	90	5	6.28	0.8	Positive
d3-4-Methylguaiacol rutinoside	467	147	90	10	6.28	0.8	Positive
4-Methylguaiacol rutinoside	464	309	90	5	6.31	0.8	Positive
4-Methylguaiacol rutinoside	464	309	90	5	6.31	0.8	Positive
4-Methylguaiacol rutinoside	464	164	90	10	6.31	0.8	Positive
4-Methylguaiacol rutinoside	464	147	90	10	6.31	0.8	Positive

Table S2. Quantify and qualify ion, and calibration curve details for GC-MS

Compounds	Quantify ion	Qualify ion	Retention time (min)	Calibration range (µg/L)	R^2
guaiacol-d3 (internal standard)	127	112	6.80		
guaiacol **1**	124	109	6.79	1.0-100	0.998
4-methylguaiacol **2**	138	123	7.78	0.25-50	0.990
4-ethylguaiacol **3**	137	152	8.52	0.1-4	0.993
p-cresol **4**	108	107	8.98	1.0-80	0.985
m-cresol **5**	108	107	9.06	1.0-80	0.997
o-cresol **6**	108	107	8.18	1.0-80	0.989
phenol **7**	94	66	8.23	2.0-130	0.993
4-ethylphenol **8**	122	107	9.89	0.1-30	0.993
syringol **9**	154	139	10.75	2.5-480	0.995
4-methylSryingol **10**	168	153	11.55	0.2-100	0.998

Table S3. Basic chemical analysis of wine samples

Wine Sample	Alc %	pH	TA (g/L)	RS (g/L)	Malic (mg/L)	AA (g/L)	FSO2 (mg/L)	TSO2 (mg/L)
High smoke-impacted	14.13	3.93	5.18	0.25	4.67	0.42	15.00	63.33
No smoke-impacted	15.25	3.95	5.11	0.34	24.50	0.38	14.17	20.50

TA= titratable acidity in g/L of tartaric acid
RS=residual sugar
AA= acidic acid in g/L acidic acid units
FSO2 and TSO2 is free and total SO2.

Table S4. Basic chemical analysis of grape samples

Grape sample	Brix	pH	TA (g/L)
High smoke-impacted berry	24.0	3.4	5.9
No smoke-impacted berry	25.8	3.7	4.54

Brix = the percentage of total solids (g) in solution (g)
TA= titratable acidity in g/L of tartaric acid

Table S5. Recovery and coefficient of variation (CV) (n=3) of spiked VPs from wine and berry homogenate.

	Wine		Acidified Wine (pH=1.0)		Berry		Acidified berry (pH=1.0)	
Compounds	Recovery	CV (%)	Recovery	CV (%)	Recovery	CV (%)	Recovery	CV (%)
guaiacol **1**	96%	7%	98%	6%	109%	8%	112%	4%
4-methylguaiacol **2**	109%	8%	123%	7%	87%	3%	85%	6%
4-ethylguaiacol **3**	82%	12%	90%	5%	74%	19%	87%	6%
p-Cresol **4**	115%	3%	106%	9%	83%	11%	125%	7%
m-Cresol **5**	117%	18%	111%	15%	102%	13%	108%	9%
o-Cresol **6**	99%	4%	108%	15%	143%	7%	146%	5%
Phenol **7**	81%	8%	93%	18%	121%	12%	140%	6%
4-ethylphenol **8**	139%	6%	133%	7%	93%	19%	127%	7%
sryingol **9**	89%	7%	104%	6%	75%	12%	86%	11%
4-methylSryingol **10**	94%	6%	97%	11%	81%	4%	80%	18%

Table S6: Mass balance of VP glycosides and free VPs produced in spike-recovery experiments. The unit for wine is µg/L and for berry is µg/kg. The values are expressed as the average ± standard deviation with N=3

Fortified VP glycosides		VPs							
				Wine				Berry	
VP glycoside	Spiked concentration	VPs generated	Expected concentration (100% recovery)	Generated concentration by acid hydrolysis	Generated concentration by *Cb*BglB-1	Generated concentration by *Aory*Rut	Generated concentration by *Cb*BglB + *Aory*Rut	Generated concentration by acid hydrolysis	Generated concentration by *Cb*BglB + *Aory*Rut
guaiacol glucoside **1a**	40	guaiacol **1**	17.34	14.5 ± 1.5	16.0 ± 1.1	0.7 ± 0.4	17.1 ± 1.1	16.7 ± 1.0	16.7 ± 1.4
guaiacol gentiobioside **1b**	40	guaiacol **1**	11.07	11.1 ± 0.5	10.3 ± 0.3	1.3 ± 0.4	11.5 ± 0.6	10.2 ± 2.2	12.0 ± 1.3
guaiacol rutinoside **1c**	40	guaiacol **1**	11.48	10.7 ± 1.4	9.3 ± 0.3	9.8 ± 1.2	10.9 ± 1.4	10.8 ± 0.4	10.5 ± 1.3
4-methylguaiacol rutinoside **2c**	40	4-methylguaiacol **2**	12.38	10.8 ± 1.9	0.2 ± 0.1	5.5 ± 0.6	9.6 ± 1.5	11.9 ± 1.0	13.1 ± 1.9
p-cresol rutinoside **4c**	40	p-cresol **4**	10.38	8.6 ± 1.2	0.6 ± 0.1	9.5 ± 0.6	10.3 ± 1.4	9.8 ± 1.3	9.8 ± 1.2
phenol rutinoside **7c**	40	phenol **7**	9.35	7.9 ± 0.7	1.6 ± 0.2	8.2 ± 0.4	9.3 ± 1.2	8.7 ± 1.3	9.3 ± 1.1
syringol gentiobioside **9b**	40	syringol **9**	12.89	11.4 ± 1.4	10.3 ± 1.3	3.2 ± 0.8	11.9 ± 1.1	11.7 ± 1.0	10.5 ± 1.7
4-methylsyringol gentiobioside **10b**	40	4-methylsryingol **10**	13.66	13.5 ± 1.5	8.5 ± 1.1	4.4 ± 1.0	12.8 ± 1.5	12.9 ± 1.9	12.1 ± 0.6

Table S7. Concentration of free VPs and total VPs after two hydrolysis methods. The unit for wine is µg/L and for berry is µg/kg. The values are expressed as the average ± standard deviation with N=3.

Sample		VPs									
		**1**	**2**	**3**	**4**	**5**	**6**	**7**	**8**	**9**	**10**
Smoke-impacted wine	Free	37.0±0.9	6.2±0.2	0.5±0.1	16.3±3.4	26.2±0.2	23.5±0.2	79.1±1.6	6.2±0.2	51.2±1.1	4.1±0.5
	After acid hydrolysis	74.4±5.1	16.3±0.9	1.2±0.1	31.7±6.7	43.1±2.1	30.8±1.9	96.4±6.0	23.0±1.2	471.2±35.7	99.5±9.9
	After *Cb*GglB-1 hydrolysis	53.0±1.6	9.6±0.4	0.6±0.1	20.3±0.8	36.2±1.3	30.2±1.1	94.0±1.8	9.1±0.4	246.9±8.3	40.0±1.3
	After enzyme cocktail hydrolysis	78.9±0.1	16.6±0.3	0.9±0.2	34.7±0.4	60.5±0.4	39.8±0.3	125.1±0.6	28.4±0.3	407.7±3.7	69.6±2.8
Non-smoke-impacted wine	Free	2.2±0.1	0.3±0.4	0.1±0.1	1.1±0.2	1.1±0.2	1.6±0.2	7.4±0.6	0.3±0.1	31.1±3.1	0.3±0.1
	After acid hydrolysis	4.7±0.1	0.5±0.4	0.2±0.1	4.2±1.9	1.9±0.1	2.8±0.1	11.9±0.3	2.9±1.0	35.9±0.9	0.8±0.1
	After *Cb*GglB-1 hydrolysis	3.3±0.4	0.5±0.1	0.1±0.1	2.7±0.2	1.8±0.2	2.9±0.2	9.6±0.9	0.7±0.2	33.3±1.8	0.6±0.1
	After enzyme cocktail hydrolysis	4.0±0.3	0.7±0.4	0.2±0.1	3.8±0.1	2.9±0.1	3.2±0.2	14.8±0.1	2.5±0.1	35.1±0.8	1.0±0.1
Smoke-impacted grape	Free	7.6±0.7	1.6±1.2	0.3±0.1	1.6±1.8	1.3±0.8	6.8±1.6	6.4±4.4	1.1±0.1	2.9±3.4	0.9±0.3
	After acid hydrolysis	43.3±4.7	34.0±4.5	1.0±0.4	16.5±3.0	7.9±1.0	12.4±1.6	31.4±4.0	5.9±1.1	184.5±25.5	36.9±1.5
	After enzyme cocktail hydrolysis	42.6±2.1	30.1±2.5	1.1±0.1	18.7±1.6	15.3±1.1	17.1±1.6	47.7±2.4	12.1±1.9	143.7±10.1	27.1±1.3
Non-smoke-impacted grape	Free	1.7±0.2	0.3±0.1	0.4±0.1	1.5±1.2	0.2±0.1	2.0±0.4	2.2±1.0	0.2±0.2	2.7±1.3	0.2±0.3
	After acid hydrolysis	5.8±0.8	3.6±0.4	0.6±0.1	1.9±0.3	1.0±0.1	2.9±0.2	8.1±0.5	1.4±0.3	20.1±2.2	1.0±0.1
	After enzyme cocktail hydrolysis	5.0±1.0	3.6±0.3	0.6±0.0	2.1±0.2	2.2±0.6	3.7±0.2	13.9±0.3	2.3±0.3	14.9±2.5	0.8±0.2

Table S8. Genes and NCBI/Uniprot identifiers encoding screened enzymes

Name	Identifiers	Sequence
*Cb*BglB-1	MBR2796233.1	MAQFPSDFIWGVACASYQCEGGWDADGKGPNIWDDFCHRAGGSTVKNNDNGDVACDSYHRYPEDIALMKQHNIRAYRFSISWARVMPDGDGALNEAGLAYYDDLVNRLLENGIEPMVTLFHWDLPSALQYRGGWLNREMVDIFARYAGVIATRFKGRVKKYMTINEPQCIALGYYTDTMAPGWRCPDEDVARVFHIIALAHSAAQRAIKAVDPEALVGLVPCGRLCYPREETPENIESAYRASFDLTQRWAFNFNIIMDSVVLRRYDDSAPEAVRRFAATIPQSDWEAMETPDFIGVNVYNGTMVDAAGNDVDCYPGFPRTACKWPITPEVMHYGPMYLYRRYGLPMIISEDGLSCNDIIFRDGQVHDPKRIDFLHRYLTELSRAIAGGVPVKGYMQWSFLDNFEWASGYDERFGLIYVDYPTLRRIPKDSARWYANVIATNGACLEEG
*Aory*Rut	A0A1S9DRB1	MAPHPRVQSPEYVNWTTFKANGVNLGGWLVQESTIDSQFWGTYSGGADDEWGLCEHLGSRCGPVLEHRYATYITERDIDKLASVGVGVLRIPTTYAAWIKLPGSQLYSGNQTAYLKQIADYAITKYGMHIIVDVHSLPGGTNGLTIGEASGHWGWYYNETAFDYSMQVIDAVISFVQNSGSPQSYTIEPMNEPTDNPDMSVFGTPAALSDRGATWVLKYIRAVIDRVASVNPNIPVMFQGSFKPEQYWSNQLPADANLVFDVHTYYFERNVTSETLPARLYADAQSKAGDGKFPVFTGEWAIQTLYQNSFALRERNVNAGLDAMYKYSQGSCYWTAKFSGNATVNGQGTQADYWNFEYFIDHGYIDLTRFHDTK
OscbBglB	MBQ3381008.1	MKQFPEQFLWGVACASYQCEGAWNEDGKGPSIWDDFCHDPAGHIRNGDTGDIACDVYHRFREDIALMKKLGIKAYRFSISWPRVIPDGDGEVNEAGLRFYDELVDELLKSGIEPLITLYHWDLPSALQDKGGWLNRDIVAAFGRYAELIAERFRGRVRRYMTINEPPCITVLGYGSGIHAPGLRLNDEKLAQIFHILALAHSEAYRRIKAVSGPETRVGIVPCGRLCYPLEDTPENREAAYRATFDLSRERWGFTFNIILDSLIFRRYDDSAPEAVKRFAATVPACEWEQMEKPDFIGINVYNGECVDAEGKAAGRWPGFPLTATKWPVTPEVMHYAPLNLSRRYGLPMMITENGQSCNDRIFRDGQVHDPERIDFLHRYLLELHKAVEEGAPLEGYLQWSFLDNFEWSEGYGERFGIVYVDYPTQRRIPKDSAFWFGRIIESNGALLFSED
CbBglB-2	MBQ3268742.1	MVKFPSDFIWGAACAAYQCEGAWNEDGKGPSIWDDFCHELGNQHVNNGDSGDVACDSYHRYREDVALMKQHGLKAYRFSISWPRVIPDGDGEVNEAGLAYYDALVDALLENGIEPMITLYHWDLPSALHLKGGWQNRQIAEWFARYARIIAERFKGRVTRYMTINEAQCITLLGYGIGVHAPGLKLPGEELARIYHNIALAHSAAQRAIKAVSPEAQVGFVPCGNLCYPVVDTPENRDAAYRASFAYTERWGFNFNIVL

		DSLVLRRYDDSAPAVLKKFAATIPASDWAQMEAPDFI GINVYQGQPVDGEGKPVPRPAGHPLTACKWPITPPVM HYGPLNVYRRYQLPIIISENGLSCNDVEFLDGKVHDPD RENYLHRYISELSRAIQDGTPVFGYLHWSFLDNFEWNS GYDERFGLIYVDYATQKRIPKDSAAWYAKVIETNGAC LNG
C*b*BglB-3	MBQ6595599.1	MAYFPKDFLWGVACASYQCEGGWDADGKGRNIWDD FCREPGKVKYGDTGDTACDTYHRIDEDVALMKKFGV QAYRFSLSWARILPEGDGEVNEAGLEYYSRVVDLLLE NGIEPMVTLYHWDLPSALQYKGGWLNRDIVKAFGRY ADIVSKRFGDRVTRYMTINEPQCITALGYGKGVLAPG WVLPDVDLARIYHNIALSHSEAQRRIRGNVPGAQVGIV PCGQLCYPKEETEENIEAAYRASFDLSHGWWAFKFNIC LDNLIRRGWDDTAPETLRRFQDTVPASDWQLMETPDF LGMNVYNGDCVDGSGRNVPQPSGHPVTGCKWPVTPE VLHYGPIHLYRRYQLPLYITENGLSCNDVVSLDGLVHD PARIDFLHRYLRELSKALQAGIPLRGYLHWSFLDNFEW ASGYDERFGLIHVDYQTLVRTPKDSAAWYRRVIETNG AEL
TcBglB	WP_088862624	MYKFPRDFVFGYSWSGFQFEMGLKGSEVPNSDWWV WVHDMENIMTGLVSGDLPENGPAYWHLYSKDHDMA EKLGMDAIRGGIEWARIFPEPTFDVRVTVERDEEGRITS VDVPESAIEELEKRANLEALEHYKRIYSDWRERGKVFI LNLYHWPLPLWLHDPIKVRRFGPDRAPSGWLDDRSVV EFAKFAAFVAYHLNDFVDSWSTMNEPNVVYENGYGR PNSGFPPGYLSFEAVEKAKLNLIYAHARAYDAIKEFSE KPVGVIYAYTWLDPLSEEIAEDVRKIRENELYSFVDSV HFGESRTVGEGREELKGRVDWLGVNYYSRIAFDRVNG HVVPLPGYGFSGVRKGYAKSGRPCSDFGWEIYPEGLE KLLRELNERYGLPMMITENGMADEADRYRSYYLVSHL RAIHSAIEAGADIRGYLHWSLTDNYEWAKGFQMKFGL LKVDWESKRRYIRPSALVFKEIATQKAIPEELSHLSDLR PLLQD
VsBglB	KJR72531	MSLKFPKDFGFGFSTAGFQHEMGLPGSEYESDWWVW VHDPENIAAGIVSGDLPENGPGYWHLYKSDHDIAFSLG MDTLRLGIEWARVFPKPTFEVNVNADIRDGSVVSVDV SEEALRRLDGLANRDAVQHYIEIIKDWKDRGGKLIVNL YHWPLPLWVHDPLVVRRSGPNNAPTGWLDPRTVVEF AKYAAYLAWRLGEFVDMWSTMNEPNVVFSNGYLYV KSGFPPGYLGIELMLRARGNLMTAHARAYDALREFSK APIGIIYAISDVQPLTKDDEEAAKAYEEAGQVSFLDAIT KGSGREDLRGRLDWLGINYYSRTVVTTAKSQSSILPPA RVVPGYGFACGPNAVSRDGRPCSDFGWELYPEGLYNV LTRYWGRYGLPIIVTENGIADARDQWRSWFIVSHLYQL HRALGQGVDVRGYLHWNLIDNYEWASGFRMKFGLV

		QVDYNTKKRYLRPSALVFREIARNKEIPEYLTHMIQSPTI
TgBglB	WP_062370819.1	MWKFPKDFLFGYSWSGFQFEMGLEGSEVPNSDWWVWVHDTENIFSGLVSGHLPENGPAYWHLYKQDHDIAEGLGMEAIRGGIEWARLFPKPTFDVKVDIEKDEDGNIVAVDVPERAIEEMEKLADMKALEHYREIYSDWKGRGKVFILNLYHWPLPLWLHDPIAVRRLGPDRAPSGWLDERSVVEFVKFAAFVAYHLNDLVDMWSTMNEPNVVYEQGYTRPNSGFPPGYLSFESSTKAARNMAQAHARAYDVIKEHSKAPVGLIYSFVWHDALNEEAEDIVKEIRKRHYEFVTAVHSGSSGLLGERPDMKGKLDWIGVNYYTRVAYRMNNGSIEVPPGYGYMCERGGFAKSGRPASDFGWEIYPEGLENILRDLHRIYGLPMMITENGIADAADRYRPYYLVSHLKAVHSAMEAGADVRGYLHWSLTDNYEWAQGFRMRFGLVHVDFETKKRYLRPSALAFREIATRKEIPEELSHLADLTPLMRD
TaBglB-1	RLG75229.1	MSLKFPKDFKFGFSEAGFQFEMGLPGSENPHSDWWTWVHDQENITAGIVSGDLPENGPGYWHLYQKDHEIADSLGMDSARLGIEWSRLFPKPTFNIKADVEKDSAGNIISVEVGEKSLEELDKIANKEAVEHYRRIFEDWRKRGKLLIINLYHWPMPVWLHDPIKVRKLGPDRAPAGWVDERSVVEFTKFAAYVAWKLGDLPDMWSTMNEPNVVYTQGYVSIKSGFPPGYLSVEASLKAAKHLIEAHARAYDVLKKMTKKPVGIIYATAEIEPLTTEDKEIAEAAYAQHNFSFMDAIFTGTSQLVGGERKDLARHLDWIGINYYSRLVVTRAKTAAGWRVVEGYGFACQPRGISRAGRPCSDFGWEVYPEGLYSVVKRFWERYRLPMLITENGIADSVDALRPRYLVSHLAQVHKLVSEGVELKGYLHWALTDNYEWAQGFRMRFGLVYVDYETKK
IaBglB	ADM27756.1	MGLKYPKEFIFGFSESGFQFEMGLPGSEDPNTDWWVWVHDPENIASTLVSGDFPENGPGYWHLYRQDHDIAERLGMDGARIGIEWSRIFSKPTFDVKVDVARDERGNIVYIDVAEKALEELDRIANKDAVNHYREILSDWKNRGKKLIINLYHWTLPLWLHDPIKVRKLGIDRAPAGWVDERTVIEFVKYVAYIAWKLGDLPDLWCTMNEPNVVYSIGYINIKIGYPPGYLSFEAASKAMKHLVEAHARAYEVLKRFTNKPVGIIYVTTYHEPLKESDRDVAEAAMYQAVFDFLDSITIGRSMSIGERKDLEKHLDWLGINYYSRLVVERYGNAWRVLPGYGFACIPGGTSLAGRPCNDAGWETYPEGLYIMLKRCWERYRLPIIVTENGTADAIDRLRPRYLATHLYQVWKALSEGVDIRGYLHWALVDNYEWSSGFRMRFGLVHVDFETKKRYLRPSALLFREIASSKEIPDEFMHMTQPQILI
TaBglB-2	RLG79985.1	MKIPKEFMLGASLSSFQFEGGFRGDEDPNNDWWIWVHDWENIIAGIVSGDFPENGPGYWRLFRQDHDLAEKLGMNTLRVGIEWSRIFPRPTFDVKVTVDKDEDGNILHVDIDEKALAKLDEIADQDAVKHYIEMYSDWKNRGKQLIINLY

		HWPLPLWIHDPIKVRKYGPDRAPSGWLDEKTIIEFVKY<br>AAYVSWKLRDLADMWSTMNEPNVVYEQGYMFIKNG<br>FPPGYLSFEAAEKAKKNLIYAHARAYEVVKKITGKPVG<br>IIYALPYIESLNGEKETLEAIKSYRIYEFLDLIIKGKSVRN<br>PILRKELASRADWLGVNYYSRIVFKFIHGKPIVLQGYGF<br>FCSSSGVSKMGLPCSDFGWEIYPQGLYLLLKEIHTRYN<br>GLPIIVTENGISDKADKLRPKYLVSHLYNTLKARNEGV<br>PVKGYLHWSLIDNYEWAQGFRQRFGLVIVDFNTKKRY<br>IRPSALVFREIALSQEIPEELMHLTHVEPLI
CmBglB	WP_012185712	MIKFPSDFRFGFSTVGTQHEMGTPGSEFVSDWYVWLH<br>DPENIASGLVSGDLPEHGPGYWDLYKQDHSIARDLGL<br>DAAWITIEWARVFPKPTFDVKVKVDEDDGGNVVDVE<br>VNESALEELRRLADLNAVNHYRGILSDWKERGGLLVI<br>NLYHWAMPTWLHDPIAVRKNGPDRAPSGWLDKRSVI<br>EFTKFAAFIAHELGDLADMWYTMNEPGVVITEGYLYV<br>KSGFPPGYLDLNSLATAGKHLIEAHARAYDAIKAYSRK<br>PVGLVYSFADYQPLRQGDEEAVKEAKGLDYSFFDAPI<br>KGELMGVTRDDLKGRLDWIGVNYYTRAVLRRRQDAG<br>RASVAVVDGFGYSCEPGGVSNDRRPCSDFGWEIYPEG<br>VYNVLMDLWRRYRMPMYITENGIADEHDKWRSWFIV<br>SHLYQIHRAMEEGVDVRGYFHWNLIDNLEWAAGYRM<br>RFGLVYVDYATKRRYFRPSALVMREVAKQKAIPDYLE<br>HYIKPPRIE
TuBglB	WP_013680114<br>.1	MRKFPSGFRWGWSGAGFQFEMGLPGSEDPNTDWFAW<br>VHDPENIAAGLVSGDFPENGVAYWHLYKQFHDDTVK<br>MGLNTIRFNTEWSRIFPKPTFDVRVHYEVREGRVVSVD<br>ITEKALEELDKLANKDAVAHYREIFSDIKSRGLYFILNL<br>YHWPMPLWVHDPIKVRRGDLSGRNVGWVAETTVVEF<br>AKYAAYVAWKFGDLADEFSTFNEPNVTYNLGFIAVKA<br>GFPPGYLSFQMARRAAVNLITAHARAYDAIRLTSKKPV<br>GVIYAASPVYPLTEADKAAAERAAYDGLWFFLDAVA<br>KGVLDGVAQDDLKGRLDWLGINYYSRSVVVKRGDGY<br>AGVPGYGFACEPNSVSRDGRPTSDFGWEIYPEGLYDIL<br>TWAWRRYGLPLYVTENGIADQHDRWRPYYLVSHLAQ<br>LHRAIQDGVNVKGYLHWSLTDNYEWASGFSKKFGLIY<br>VDLSTKRHYWRPSAYIYREIASSNGIPDELEHLEKVPV<br>ASPEVLRGLRSL
CmBglB	PSN97385	MISLPGIRFGWSQAGFQSEMGLPGSEDPNSDWFAWVH<br>DKENIAAGVVSGDLPEYGPAYWHRFREFHDAAERMEL<br>KIARIGVEWSRVFPKPTLDVQVDIEQRGDMVTHVDVS<br>QSQLEKMDAIASKDAVEHYRTIFSDLKRRGIEFVLNLY<br>HWPLPLWIHDPVAVRRGEKTERTGWLSTRTVVEFAKF<br>AAYISWKLDDLVDAYSTMNEPNVVWGAGYTSVKSGF<br>PPGYLSFAHSSRAMYNMVQAHARAFDVLKTHKKPVGI<br>IYANSDFQGLTAGDADVASKAEFDNRWRFFEAIVNGD<br>LGGYRDDLKGRLEWIGVNYYTRSVVRKAGEGYVVVR

		GYGHACERNSLSADGRPTSDFGWEFYPEGLGNVLVKY<br>REKYGLPLYVTENGIADEADYQRPYYLVSHIYQVYQA<br>LRRGADVKGYLHWSLADNYEWASGFTPRFGLLRVDY<br>TNKSLFWNPSAFVYKEIAGSNGIPDQLEHLNRVPPTRG<br>LRR
FcBglB	WP_090223355	MFPNSFMFGASLSGFQFEMGNPSDPSELDTQTDWFVW<br>VRDLENLLNGIVSGDLPESGAGYWKSYEKIHQLAVDF<br>GMDTLRIGIEWSRIFPSSTREIPFGEGMLEKLDSIANKD<br>AVEHYRKIMEDMKSKGLKVFVNLNHFTLPLWLHDPL<br>AVRKGKPTDKLGWVSDDAPVEFAKYAEYIAWKFGDI<br>VDYWSSMNEPHVVAQLGYFQILAGFPPSYFNPEWYIK<br>SLRNEATAHNLTYDAIKRHTDKPVGVIYSFTWYDTLKP<br>NNSEIFENAMWLANWNFMDQVKDKVDYIGVNYYTR<br>AMIDKLPKPIEIQDFELNWYVVRGYGYACQEGGFALS<br>GRPASEFGWEIYPEGLYYLLKAIYERYNKPLIVTENGIA<br>DQNDKYRAQVLISHLYAVEKAMNEGVDVRGYLHWSI<br>VDNYEWAKGYSKRFGLAYTDFEKKLYIPRPSMYVFRE<br>IAKTRSIDQFKGYDPYGLMKF
FtBglB	WP_069292479	MFPKDFMFGVSMSGFQFEMGWGDERDLDPNTDWFV<br>WVREPGNLVNGVVSGDLPEFGAGYWLNYEKIHQLAV<br>DFGMDTIRIGIEWSRIFPTSTESVDVRDPNFLDKLDELA<br>NKKAVEHYRKIMEDIKSKGLKLFVNLNHFTLPLWLHD<br>PVAVHYGRPTDKLGWVSERTVHEFAKYVAYMAKYG<br>DIVDLWSTMNEPHVVSQLGYFSVSAGFPPAYFNPEWYI<br>LATKHLAMAHNLGYDMIKRFSDKPTGVIYSFTWYDTL<br>NPNDREILEEAMYLTNWFFMDMVKEKLDYVGVNYYT<br>RTVIDRVEQPLAMGNFNVRWRILKGYGYACDEGGVA<br>LSGRPASDFGWEMYPEGLYYVLKAVSERYSKPIIVTEN<br>GVADWNDRLRSTHLISHLYYVERALEDGIDVKGYLH<br>WSIVDNYEWAKGYSKRFGLAWTNFQTKTYHPRPSMYI<br>FRDIIRARTTKEFIGFDPYKVRTEL
FgBglB	WP_072757753	MFPKDFMFGASLSGFQFEMGNPNDPKEVDPNTDWFV<br>WVREPENLVNGIVSGDLPEYGAGYWKNYEKVHQLAV<br>DFGMDTLRIGIEWSRVFPTSTREVPTGDGMLEALDKIA<br>NKEAVEHYRKIMEDMKSKGLKVFVNLNHFTLPLWIHD<br>PISVHKGIPTDKLGWVSDDTPIEFAKYAEYIAWKFSDIV<br>DYWSSMNEPHVVAQLGYFQILAGFPPSYFRPEWYIKSL<br>VNEAKAHNLAYDAIKKYTSRPVGIIYSFIWYDTVNPQD<br>RDIFENAMWLTNWYYIDMVKDKADYIGINYYTRSLID<br>RLPASGMKFGDFELNWYPLRGYGYACPEGGMSLSGRP<br>ASEFGWEVYPEGLYNLIKAIYERYKKIIIVTENGIADEK<br>DKYRSHYLISHLYAVEKAMNEGANVIGYLHWSIVDNY<br>EWAKGYSKRFGLAYTDLEKKIYVPRPSMYIFREIAKTK<br>SIEQFKDYDPYKLMKF
GfBglB		MNATDCITHFPKDFIWGAACASYQCEGAWNEDGKGPS<br>IWDEFCHDTIDGKNLNISNGDIASDFYHHWREDIALMK

		AHNIRAYRFSVSWSRVLPDGEGKVNEQGLQWYSDVV DELLANGIEPMITLYHWDLPAALQDKGGWLNRDIIDVF AEYAAIIAEKLKGRVKRYMTLNEPACIVQAGYSKMLH APGWRVSDEKMARIFHILALSHSAAKRAIKMIDPAAQV GIVTCGRLFWPERDTPENREAAYRASFDLSDAYWPFK HNILLDSLIFCRYDASIPAPVRRFAATIPESDWERMETP DFIGINVYEGPCINAARETVAPMYGSPVSACRWPITPEV LHYGPEYIYRRYRLPVLISENGISCNDMIFDDGRVHDPQ RIQYLRRYLLALDKAIEEGTPVMGYLQWSVMDNMEW NSGYNERFGMFFVDYQTKQRIPKDSAAWYAKVIATNG QSLGEMPRF
TpBglB		MALKFGKEFKFGFSTVGVQHELGLPGSEFESDWIAWL RDPENIASGLVSGDDPFSGPGYWHLYREDHAIAEYLG MNAAWITVEWARIFPKPTTEVRAYVEQDGERITQVSLE ESELERLLRLANREALSHYREIMSDWKSRGGFLIVNLF HWSLPLWLHDPVAVRSRGPDRAPSGWLDKRTVVEFA KFAALVARELDDLADAWYTMNEPMVVARLGYVSVSS GFPPGYLSLKAYEEAKVRLAEAHARAYDALREVSGKP VGLVESVSPVTVLGGESSLAELVLREQLAVLDAARFGT VGGEVREDLGGRLDWVGVNYYTRVVVSPGGPLGFRV ESGYGYSCAPRGVSRDGRPCSDVGWEVYPEGLFEAISL VSKRYGLPVYITENGVADSRDALRPSFIVSHLYQVARL LEQGVDVRGYFHWNLTDNLEWAKGFSPRFGLVEVDY QTKKRRLRPSALVFREIALSREVPYEVALAGEWS
SaciBgl	P14288	MLSFPKGFKFGWSQSGFQSEMGTPGSEDPNSDWHVW VHDRENIVSQVVSGDLPENGPGYWGNYKRFHDEAEKI GLNAVRINVEWSRIFPRPLPKPEMQTGTDKENSPVISVD LNESKLREMDNYANHEALSHYRQILEDLRNRGFHIVL NMYHWTLPIWLHDPIRVRRGDFTGPTGWLNSRTVYEF ARFSAYVAWKLDDLASEYATMNEPNVVWGAGYAFPR AGFPPNYLSFRLSEIAKWNIIQAHARAYDAIKSVSKKSV GIIYANTSYYPLRPQDNEAVEIAERLNRWSFFDSIIKGEI TSEGQNVREDLRNRLDWIGVNYYTRTVVTKAESGYLT LPGYGDRCERNSLSLANLPTSDFGWEFFPEGLYDVLLK YWNRYGLPLYVMENGIADDADYQRPYYLVSHIYQVH RALNEGVDVRGYLHWSLADNYEWSSGFSMRFGLLKV DYLTKRLYWRPSALVYREITRSNGIPEELEHLNRVPPIK PLRH
CmaqBgl	A8MBR0	MDISFPKSFRFGWSQAGFQSEMGTPGSEDPNTDWYVW VHDPENIASGLVSGDLPEHGPGYWGLYRMFHDNAVK MGLDIARINVEWSRIFPKPMPDPPQGNVEVKGNDVLA VHVDENDLKRLDEAANQEAVRHYREIFSDLKARGIHFI LNFYHWPLPLWVHDPIRVRKGDLSGPTGWLDVKTVIN FARFAAYTAWKFDDLADEYSTMNEPNVVHSNGYMW VKSGFPPSYLNFELSRRVMVNLIQAHARAYDAVKAISK KPIGIIYANSSFTPLTDKDAKAVELAEYDSRWIFFDAIIK

		GELMGVTRDDLKGRLDWIGVNYYSRTVVKLIGEKSYVSIPGYGYGCERNSISPDGRPCSDFGWEFYPEGLYDVIMKYWSRYHLPIYVTENGIADAADYQRPYYLVSHIYQVYRAIQEGANVKGYLHWSLTDNYEWASGFSMRFGLLQVDYSTKKQYWRPSAYVYREIAKSKAIPEELMHLNTIPPTRSLRR
TvolBgl		MVENNFPEDFKFGWSQSGFQSEMGYDNAMDDKSDWYVWVHDKENIQSGLVSGDMPENGPGYWNNYKSFHEAAQNMGLKMARIGVEWSRLFPEPFPEKIMADAKNNSLEINNNILSELDKYVNKDALNHYIEIFNDIKNRNIDLIINMYHWPLPVWLSDPVSVRKGIKTERSGWLNDRIVQLFALFSSYIVYKMEDLAVAFSTMNEPNVVYGNGFINIKSGFPPSYLSSEFASKVKNNILKAHSLAYDSMKKITDKPVGIIYANTYFTPLDPEKDNDAIAKADSDAKWSFFDPLIKGDKSLGINGNKLDWIGINYYTRTMLRKDGDGYISLKGYGHSGSPNTVTNDKRPTSDIGWEFYPEGLEYVIMNYWNRYKLPMYVTENGIADNGDYQRPYYLVSHIASVLRAINKGANVKGYLHWSLVDNYEWALGFSPKFGLIGYDENKKLYWRPSALVYKEIATKNCISPELKHLDSIPPINGLRK
PfurBgl	E7FHY4	MKFPKNFMFGYSWSGFQFEMGLPGSEVESDWWVWVHDKENIASGLVSGDLPENGPAYWHLYKQDHDIAEKLGMDCIRGGIEWARIFPKPTFDVKVDVEKDEEGNIISVDVPESTIKELEKIANMEALEHYRKIYSDWKERGKTFILNLYHWPLPLWIHDPIAVRKLGPDRAPAGWLDEKTVVEFVKFAAFVAYHLDDLVDMWSTMNEPNVVYNQGYINLRSGFPPGYLSFEAAEKAKFNLIQAHIGAYDAIKEYSEKSVGVIYAFAWHDPLAEEYKDEVEEIRKKDYEFVTILHSKGKLDWIGVNYYSRLVYGAKDGHLVPLPGYGFMSERGGFAKSGRPASDFGWEMYPEGLENLLKYLNNAYELPMIITENGMADAADRYRPHYLVSHLKAVYNAMKEGADVRGYLHWSLTDNYEWAQGFRMRFGLVYVDFETKKRYLRPSALVFREIATQKEIPEELAHLADLKFVTRK
TgorBgl		MYKFPRDFLFGYSWSGFQFEMGLPGSEVPNSDWWAWVHDIENIAAGLVSGDLPENGPAYWDLYKKDHDIAESLGMDAIRGGIEWARIFPKPTFDVKARVERDEKGNIVSVEVPESSIKELEKIADMNALEHYREIYADWKERGKTFILNLYHWPLPLWLHDPLKVRKLGPDRAPAGWLDDKSVVEFAKFAAFVAYHLDDLVEVWSTMNEPNVVYQNGYTRPTHGFPPGYLSFEAERKAKMNLIQAHARAYDVIKEYSDKDVGVIYAYTWPDPLREDIEEEVRAIRERELYSFVDAVHFGKAADVEERDDLKGRVDWLGVNYYSRIAFDMVNGHVLPVPGYGFSGERGGYARSGRPCSDFGWEIYPEGLEQLLKDLAKRYGLPMMITENGIADAADRYRPHYLVSHLKAVHEAMKEGADVRGYLHWSLTDNYEWAQGFRMRFGLVYYDMETKKRYLRPSALVFREIATRKEIPEELEHLSSLDFLVRR

FnodBgl	A7HNB8	MMFPKDFLFGVSMSGFQFEMGNPQDAEEVDLNTDWY<br>VWVRDIGNIVNGVVSGDLPENGSWYWKQYGKVHQL<br>AADFGMDVIRIGTEWSRIFPVSTQSVEYGSPDMLEKLD<br>KLANQKAVSHYRKIMEDIKAKGLKLFVNLYHFTLPIW<br>LHDPIAVHKGEKTDKIGWISDATPIEFAKYAEYMAWK<br>FADIVDMWASMNEPHVVSQLGYFAINAGFPPSYFNPS<br>WYIKSLENEAKAHNLSYDAIKKYTNNPVGVIYSFTWY<br>DTVNKDDKESFENAMDLTNWRFIDMVKDKTDYIGVN<br>YYTRAVIDRLPTTIDFGEFKMNWYTLRGYGYSCEEGG<br>FSLSGRPASEFGWEIYPEGLYNILIHVYNRYKKDIYVTE<br>NGIADSKDKYRSLFIISHLYAIEKALNEGIPIKGYLHWSI<br>IDNFEWAKGYSKRFGLAYTDLSTKKYIPRPSMYIFREII<br>KDKSIDKFKGYDPYNLMKF
TafrBgl	B7IGM4	MFSKDFLFGASLSGFQFEMGNPNNEEELDKNTDWFVW<br>VRDLGNIINGKVSGDLPEYGAGYYTNYKAVHNLAKEF<br>GMNALRIGIEWSRIFKESTKDISLDDPNMLEKLDQLAD<br>KKAIEHYRDVLEDIKSKGLVAIVNLSHFTLPLWLHDPIN<br>VHKGKETEKLGWVSDDAPIEFAKYAEYIAWKFKDIVD<br>MWSSMNEPHVVSQLGYFQTSAGFPPSYFNPSWYLKSL<br>ENQALAHNLAYDAIKKHTGKPVGVIYSFTWYDTVNN<br>DEEIFESAMFLNNWNYMDRVKDKIDFVGVNYYTRAVI<br>DRLLVPIKIDNYELNWYTLSGYGYSCVEDGFANSKRPS<br>SEIGWEIYPEGLYNILKEIYNRYGKQIYITENGIADSSDK<br>YRSFYIISHLYAVEKAINEGVPVKGYLHWSIIDNYEWA<br>KGYGKRFGLAYTDFERKTYIPRPSMYILREIIKERSIDKF<br>KGYDPYGLMNF
LcasBgl		MTIQFDADFVWGAATSGPQAEGTFHKKHENIFDYHYH<br>TRPQDFYHNVGPDVASNFYNDYENDLALLKQAGVQA<br>LRISIQWTRLIDDLEAGTVDPVGADYYRRVFKTMHQL<br>GITPYVNLHHFDLPVTLQHQYGGWQSKHVVDLYVKF<br>ATRCFELYSDQVTHWFTFNEPKVIVDGQYLYQFHYPNI<br>VDGRLAVQAAYNLNLASAKAVAAFRQINRQSQGTIGT<br>IVNLTPVYPASQAPEDLAAARFAEQWANDLYLEPAIHG<br>RFPEELVARLKRDGVLWEATSDELAVIAANRIDVLGV<br>NYYHPFRVQAPAVSPDSLQAWLPDIYFDNYDMPGRK<br>MNLDKGWEIYPDALYDIAMTIKRRYDNLPWFVAENGI<br>GVANEERFLKDGMVQDDYRIQFMTDHLRFLSQAITEG<br>ANCHGYFVWTGIDCWSWLNAYKNRYGLIRNDLCNQT<br>KSLKKSGHWFSQVAATGLVAPTLRPFESEEKNHG
SequBgl		MKQSKRRYQFPEGFLWGSSTSGPQSEGTVSGDGKGPS<br>NWDYWFSLEPDKFHHQIGPEVTSTFYTNYKSDIALLKE<br>TGHTAFRTSIQWSRLIPEGVGQVNPKAVAFYREVFQEI<br>MAQDIKLIVNLYHFDLPYALQGKRGWEAKETVWAYE<br>TYAKTCFELFGDLVDTWITFNEPIVPVECGYLGHYHYP<br>CKVDAKAAVQVAYHTQLASSLAIKACHELYPKHRISIV<br>LNVTPAYPRSDQPEDVKAARIAELFQTKSFLDPSVLGV

		YPEELVVLLEAADLLPQYSADELAIIKNNPVDFLGVNY YQPLRVQAPSKTRQDGEPITLASYFEPYDMPGKKVNPH RGWEIYEQGLYDIALNLKEHYGNIDWLVTENGMGVE GEEAFLVDGQIQDDYRIAFIEDHLIQLHRALEEGANCK GYLLWTFIDCWSWLNAYKNRYGLVALDLETQKRTLK KSGHWFKTLSQTNGFDK
CbeiBgl	C8W8S6	MQYQLPKDFFFGGAMSGPQTEGRWQDDGRIPSIWDT WSNLDITAFHNRVGSYGGNDFSSRMEEDFELLKSIGM DSVRTSIQWSRLLDIDGNLNPEGERYYHQLFATAKKV GIEIFVNLYHFDMPEYLFNRGGWESREVVEAYAHYARI AFETFGKEIRYWFTFNEPIVEPEMRYTVGGWFPFVKNY SRARAVQYNISLAHALGVREYRRAKAAGFMLEDSRIG LINCFAPPYTKDNPSEADLEALRMTDGVNIRWWLDLV TKGELPQDVIDTLQSRGVDLPIRPEDKLILADGVVDWL GCNYYHPERIQAPAKDTDENGIPNFADPYVWPEAEMN VSRGWEIYPQGLYDFAMKVRDEYPELEWFVSENGMG VEREDLKKDENGVIQDDYRVDFVRRHLEWIARAIQDG AKCRGYHYWAIIDNWSWANAFKNRYGFIEVDLEDNY NRRLKKSAKWLKQIATTHIVD
CaurBgl	A9WDK4	MQQFAFPTGFLWGAATSAHQVEGNNINSDSWVLEHLP DTIYAEPSGDACDYYHRYPEDIALLAQLGFNAYRFSIE WARIEPEEGEFSFASLEHYRRMLATCHEHGLKPVVTLH HFTSPRWLIRAGGWLDPKTPDRFVRYCERVVHYLGDL IAGACTFNEPNLPVLLSKIMPASPLASPFWRAAAAEFA VTPDRLGIFQFVSQPRMREIIFAAHRRAFEVLHDGPGSF PVGMTLALVDIHAGPDGERMAAEFRRELAEVYLEQLR EDDFVGVQTYSRLVVGPAGIIPPGDDVEKTQTGEEYYP EAIGGTIRHAAAVAGIPVVVTENGLATTDDTRRVEYFR RALRSVAECLIDGIDVRGYFAWSALDNFEWISGYKPKL GIIAVDRTTQARTPKPSAYWLGNVARFNYCVFD
CrumBgl-2		MSFTKGFLIGASTAAHQVEGNNIHSDYWAQEHMPHSS FTEPSGIACDHYNRFEEDIRLMAKAGLNAYRFSIEWARI EPEEGQFDESELEHYRKVVRCCRKNGIEPLITLMHFTSP VWLIRQGGWEAESTVEYFRRYADFIVKNLGSEIKYICTI NEANMGLQLAAIAKRFQLMAQQAQKSAKNAEGTVQV GMNFQKMMENMKYAAQENAEIFGTPQPQIFVSSRTEQ GDTLVFRAHQAAKEAIKAINPDIQVGITLSLHDLQALP GGEAFAEKAWDEEFRHYLPFIQDDDFLGVQNYTRTQY GPKGQMPSPENAELTQMDYEFYPEALEHVIRSVHRDF KGNLIVTENGVATSDDTRRIEFIRRALQGVEHCLNDGIP VKGYCHWSLMDNFEWQKGYAMTFGMIAVDRTTLKR TPKESLQFLGSMIS
BdenBgl		MRETYEFPQEFIWGASTAAHQIEGNNVASDWWAREH AECADLSEPSGDAADSYHRYGEDIRMLADAGLGMYRF SIEWARIEPAEGCFSKAQLLHYRHMIDACHENGIEPMV TLNHMTLPLWLAVKGGWLNDGAVDYFDRYVRYLMPI

		LHDVTWVCTINEPNMVALTRGGTEGSDFVSASLPAPD<br>LDISAALVEAHREARGILSENPRIKSGWTIACQAFHAM<br>PGCEQEMEEYQYPREDYFTEAAAGDDFIGVQAYLRTFI<br>GKDGPVPVPEDAERTLTGWEYFPPALGIAIRHTWNVA<br>GHTPIIVTENGIATADDRRRIDYTFGAIAGMHDAMADG<br>VDVRGYLHWSLLDNYEWGSFAPTFGLACWDKDTFER<br>HPKPSLNWLGMIAKTGVMSR
SrocBgl		MTRTSLPFPDGFLWGASTAAHQIEGNNVNSDWWRKE<br>HDPAANIAEPSLDACDSYHRWEQDMDLLAELGFTDYR<br>FSVEWARIEPVPGTFSHAETAHYRRMVDGALARGLRP<br>MVTLHHFTVPQWFEDLGGWTADGAADLFARYVEHCA<br>PIIGKDVRHVCTINEPNMIAVMAGLAKTGDQGFPPAGL<br>PTPDEETTHAVIAAHHAAVKAVRAIDPDIQVGWTIANQ<br>VYQALPGAEDVTAAYRYPREDVFIEAARGDDWIGVQS<br>YTRTKIGADGPIPAPEDAERTLTQWEYYPAAVGHALR<br>HTADVAGPDMPLIVTENGIATADDARRVDYYTGALEA<br>VSAALEDGVNIHGYLAWSALDNYEWGSYKPTFGLIAV<br>DPVTFERTAKPSAVWLGEMGRTRQLPRAER
CaceBgl	Q97M15	MKFPKDFFLGAASASYQVEGAWNEDGKGVSNWDVFT<br>KIPGKTFEGTNGDVAVDHYHRYKEDVKLMAEMGLDS<br>YRFSVSWPRIIPDGDGEINQKGIEFYNNLIDECLKYGIVP<br>FVTLYHWDMPEVLEKAGGWTNKKTVDAFVKYAKAC<br>FEAFGDRVKRWITFNETIVFCSNGYLSGAHPPGITGDV<br>KKYFQATHNVFTAHARSVIEYKKLKQYGEIGITHVFSP<br>AFSVDDKEENKAAAYHANQYEITWYYDPILKGKYPEY<br>VIKNIEKQGFLPDWTDEELNTLREAAPLNDFIGLNYYQ<br>PQRVIKNHDTGEKIERTRENSTGAPGNASFDGFYRTVK<br>MDDKTYTKWGWEISPESLILGLEKLKEQYGDIKIYITE<br>NGLGDQDPIIEDEILDMPRIKFIEAHLRAIKEAISRGINLK<br>GYYAWSVIDLLSWLNGYKKQYGFIYVDHKHNLDRKK<br>KLSFYWYKKVIEERGKNI
SterBgl	D1AQN8	MERLPEDFIFGAATAAFQAEGAVNEDGRGKCYWDEY<br>LHRAESTFNGDTASDFYHKYREDTALCREYGINGIRISI<br>AWTRIIPDGSGKVNQKGIDFYNDMINACLEAGVEPYVT<br>LHHFDTPLELFKNGDWLNRENTEHFVRFAKICFENFGD<br>RVKKWITINEPWSVVAGQYIIGHFPPNIKYDVPKAVQA<br>MHNMCTAHAKAVIEYKKMNLNGEIGIIHILESKYPISE<br>KPEDIRAALLEDTLANKFMLDASLKGSYSESTMQIILEI<br>LEKYDAKLDINEDEPDILRKGAELNDFLGVNYYASHFL<br>KGYEGETEIYHNGTGKKGTSIFRIKGVGERVKNPEIETT<br>DWDWPIYPKGLYDMLVRIKNEYPDCQKLYVTENGMG<br>YKDEFINGKIEDIPRIDYIKKHLAAINQAITAGVNVKGY<br>FVWSLMDVLSWTNGFNKRYGLFYVDFQTQKRYPKKS<br>AYWYKETAESKVIK
LrhaBgl	Q29ZJ1	MRKQLPKDFVIGGATAAYQVEGATKEDGKGRVLWDD<br>FLEKQGRFSPDPAADFYHRYDEDLALAEAYGHQVIRLS

		IAWSRIFPDGAGAVEPRGVAFYHRLFAACAKHHLIPFV TLHHFDTPERLHAIGDWLSQEMLEDFVEYARFCFEEFP EIKHWITINEPTSMAVQQYTSGTFPPAETGHFDKTFQAE HNQIVAHARIVNLYKSMGLDGEIGIVHALQTPYPYSDS SEDQHAADLQDALENRLYLDGTLAGDYAPKTLALIKEI LAANQQPMFKYTDEEMAAIKKAAHQLDFVGVNNYFS KWLRAYHGKSETIHNGDGSKGSSVARLHGIGEEKKPA GIETTDWDWSIYPRGMYDMLMRIHQDYPLVPAIYVTE NGIGLKESLPAEVTPNTVIADPKRIDYLKKYLSAVADAI QAGANVKGYFVWSLQDQFSWTNGYSKRYGLFFVDFP TQKRYVKQSAEWLKQVSQTHVIPE
BthuBgl		MSKVIFPKGFLWGGAIAANQVEGAYVEDGKGLTTVDL LPTGENRWDIMKGNIHSFTPVEGEFYPSHEAIDFYHRY KEDIALFAEMGFKALRVSIAWTRIFPNGDDEKPNEAGL QFYDNLFDELLKHDIEPVVTMAHFDVPIHLVEKYGSW RSRKLVDFFETYAKTIFNRYKDKVKYWMTFNEINMLL HLPFMGAGLAFKEGDNKKQIQYQAAHHQLVASALAV KACHEIIPDAKIGCMLAAGATYPYTCNPDDIQRAMEQD RESFFFIDVQARGAYPGYAKRFFTDNNVTIEMEKEDEA ILKEHTVDYIGFSYYASRATSTDPEVLKSITSGNVFGSV ENPYLEKSEWGWTIDPKGFRITANQLYDRYQKPLFVV ENGLGAIDQLNDEDEVNDAYRIDYLEKHMIEMSEAIQ DGVDIIGYTSWGPIDLVSASTGEMKKRYGYIYVDKDN EGKGSLKRSKKDSFNWYKEVIATNGGSLES
BamyBgl		MKRFPDGFLWGGATAANQIEGAYKEGGKGLSTADVS PDGIMSPFHETDDALNLYHDAIDFYHRYQEDIALFAEM GFKAFRTSIAWTRIFPNGDETEPNEEGLQFYDRLFDELR KHQIEPVVTISHYEMPLGLVKNYGGWRNRRTVDFYER YARTVFTRYKDKVKYWMTFNEINVVLHAPFTGGGLIF REGENKQNTMYQAAHHQFVASALAVKAGHEIIPDSQI GCMIAATTTYPMTPKPEDVYAALQKERSTLFFSDVQA RGSYPGYMKRFFKENGITIEMKEGDEALLKEHTVDYIG FSYYMSMTASTAPEDLAQSKGNLLGGVKNPYLKSSEW GWQIDPKGLRITLNTLYDRYQKPLFIVENGLGAVDQPE EDGSIQDDYRINYLRDHLIEAREAIEDGVDLIGYTSWGP IDLVSASTAEMKKRYGYIYVDRGNDGKGTFERKKKKS FYWYKDVIATNGESL
CrumBgl-7		MVKQFPPGFLWGGATAANQCEGAYDADGRGLSSVDV VPYGPERMKVSRGERKMLRCEEGFSYPSHEAIDLYHH YKEDIVLFAEMGFKCYRMSVAWTRILPNGDDDIPNEA GLKFYEDVFRECRRYGIEPLVTIDHFDTPIALIEKYGGW RDRRMIDAYIKYCTALFTRYKDLVKYWITFNEINMLLH MSFMGAGIYFEPGEDKEQVKYTAANNELLASARAVKL AHELMPGSMVGCMLAAGQFYPYSCNPADIWDGLEKD RDNYFFIDVQARGYYPVWAKKRMERAGIRLELSPEDE AVLREGTVDYVAFSYYCSRCTTADPEIFEAHKRPGNA

		VFASVENPHLPFTEWGWQIDPTGLRVTINTLYDRYQKP<br>LFVVENGMGANDTLEPDGTVHDPYRIEYLRRHIEAMR<br>DAVTEDGIPLLGYTAWGCIDLVSASSGEMKKRYGMIY<br>VNKDDRGGGDLSRHRKDSFYWYKKVIASNGADLD
LlacBgl	Q9CFL0	MTFKTDFLWGGATAANQLEGAYDIDGKGLSVADAMP<br>GGKERLAILASPEFDWTIDTEHFTYPNHDGIDHYHHFK<br>EDIALFAEMGFKAYRFSVAWSRIFPKGDETTPNEKGLL<br>FYDQLIDECLKYRIEPVITISHYEMPLNLAKSYGGWKN<br>RELIEFYVRFAKVLLERYQDKVKYWMTFNEINSATFFS<br>GLSQGLVPSNGGDDKTNVFKAWHNQFVASAQAVKFG<br>HDLNKNLKLGCMSIYSTTYSFDANPVNQLATQESIQEF<br>NYFCNDVQVRGAYPAFTNRLHRKHGVNSEVLEISEED<br>LKIIAEGTVDYIGFSYYMSTVESKTGEGVQASGNMVLG<br>GVKNPFLKESEWGWAIDPDGLRYALNDLYGRYQIPLFI<br>VENGLGAIDKVEEDGTIQDDYRIDYLKKHIQSMSEAVE<br>DGVELMGYTPWGCIDLVSASTGEMSKRYGFIYVDLDD<br>SGNGTNKRFKKKSFDWYKQVIDSNGTNL
CrumBgl-6		MFKEDFLWGGATAANQFEGAWDVDGKGPSIPDHCTN<br>GTRERSKLFTQTINPEYLYPSHKASDFYHHYKEDIALL<br>AEMGYKCFRMSINWSRIFPTGMEKTPNEKGLEFYDKV<br>FDECRKYGIEPLVTLSHYEMPLALGVEKDGWLSRETID<br>CFMRYVETVFARYRDKVRYWITFNEINAGQMPIGDIIS<br>TGMVKGYEGPINGIRRTEQERYQALHHQFVASARTVR<br>LAHKKYPQFKVGNMLTFIAAYPVNCDPDNILLAAKYM<br>QNMNWYCSDVQVKGAYPYYATAMWRDRDVILNITA<br>KDIEDLENGTVDFMTFSYYMSICVGKEGEKDKVSGNL<br>TGGFKNPYLESSDWGWQIDPVGIRYALNAAYDRYRIP<br>LMIVENGLGAFDKVEEDGSVHDDYRIDYMRRHIRQMK<br>LATEDGVELMGYTNWGCIDLVSLTTGEMRKRYGQVF<br>VDKYDDGTGTLKRSRKDSFFWYRNVIRTNGMEI
Ent7Bgl		MSSREKKQLSSMPNDFLWGGAISATQVEGAYNHDGK<br>GLSNLDLALRCKKGEKRQITQQVDVNQYYPSHRAIGF<br>YESYQKDIQLFADMGFKSLRFSIQWSRIFPTGEEERPNE<br>AGLLFYEKILDELERHRIEPIITISHFDLPENLVTKYGSW<br>KNRQVITFYLRFCEALFQRFSDRVRYWIPFNEINVITYM<br>PYFSTGIHTENYQEIFQMAHHQLVASAKAVQLGRKYSS<br>NYRFATMLMYGPTYPHNCHPESVFQAMMDDEETYYF<br>GDIQIRGYYSPWAKKMLEQLGVQLAITEEDEQDLREG<br>VVDFVSISYYMSWTTAPETAAGNMATGGKNPFLEQSE<br>WGWQVDPLGLRISLNRLYQRYEKEIMIVENGLGAVDH<br>CSENGEIYDDYRIDYLQQHLLAVKQAIVLDGVPVIGFT<br>VWSAIDSISASTGEIGKRYGLIYVDLDDEGQGTLARKK<br>KASFYWYQKIIESNGAEL
GkauBgl-2	Q5KXG4	MSQQRKSIIPDDFLWGGAVTSFQTEGAWNEGGKGLSI<br>VDARPIPKGHSDWKVAVDFYHRYKEDIALFKELGFTA<br>YRTSIAWTRIFPDGEGEPNEAGLAFYDAVFDELRANGI

		EPVITLYHFDLPLALAKKYNGFASRKVVDLFERYARTV FERYRGKVNYWLTFNEQNLVLEQPHLWGAICPEDEDP EAFAYRVCHNVFIAHAKAVKALREIAPEAKIGGMVTY LTTYPATCRPEDALANVQAKELFIDFFFDVFARGAYPR YVTNQLEKKGICLPLEAGDEELLRSQTVDFLSFSYYQS QIVRHQEQDERIIKGLEPNPYLPKTKWGWAIDPIGLRIA LKDVYARYEMPIFITENGIGLEEELNENGTVDDDERID YLRRHIEQMKMAMEEGVEVIGYLMWGATDLLSSQGE MRKRYGVIFVNRDDENLRDLKRYKKKSFYWFQRVIRT NGEEL
GeoYBgl		MKYTQLKPFPTGFLWGGSTSAYQVEGAWNEDGKGPS VIDMAKHPEGTTDFKVASDHYHRYQEDIALLAEMGFK AYRFSIAWTRIYPNGEGEVNPKGLEFYNNLINEIVRHGI EPIVTIYHFDLPYALQTKGGWSNRATIDAFVNYCRTLF EHFGDRVKYWLTINEQNMMILHGEAIGIVDPDSENPKK ELYQQNHHMFVAQAKAMALCHEMLPDAKIGPAPNIA TIYPASSKPEDVLAANTYSAIRNWLYLDMAVYGRYNP TAWAYLEEKGYTPTIADGDMDILQNAKPDFIAFNYYTS QTVAASVGNESDIGHTGDQHITIGEPGVYKGASNPNLP KNDFGWEIDPIGFRTTLREIYERYRLPLIVTENGLGAYD RLEEGDIVNDTYRIDFLRNHIEQMRLAITDGVDVFGYC PWSAIDLVSTHQGISKRYGFIYVNRDEFDLKDLRRIRK QSFYWYQRVISSNGEQLD
GkauBgl-3	Q5KUY7	MEHRHLKPFPPGFLWGAASAAYQVEGAWNEDGKGLS VWDVFAKQPGRTFKGTNGDVAVDHYHRYKEDVALM AEMGLKAYRFSVSWSRVFPDGNGAVNEKGLDFYDRLI EELRTHGIEPIVTLYHWDVPQALMDAYGAWESRRIIDD FDRYAVTLFQRFGDRVKYWVTLNEQNIFISLGYRLGLH PPGVKDMKRMYEANHIANLANAKVIQSFRHYVPDGKI GPSFAYSPMYPYDSRPENVLAFENAEEFQNHWWMDV YAWGMYPQAAWNYLESQGLEPTVAPGDWELLQEAK PDFMGVNYYQTTTVEHNPPDGVSEGVMNTTGKKGTS TSSGIPGLFKTVRNPYVDTTNWDWAIDPVGLRIGLRRI ANRYRLPILITENGLGEFDTLEPDDIVNDDYRIDYLRRH IQEIQRAITDGVDVLGYCVWSFTDLLSWLNGYQKRYG FVYVNRDDESEKDLRRIKKKSFYWYQRVIATNGAEL
PchrBgl	Q25BW5	MSAAKLPKSFVWGYATAAYQIEGSPDKDGREPSIWDT FCKAPGKIADGSSGDVATDSYNRWREDVQLLKSYGVK AYRFSLSWSRIIPKGGRSDPVNGAGIKHYRTLIEELVKE GITPFVTLYHWDLPQALDDRYGGWLNKEEAIQDFTNY AKLCFESFGDLVQNWITFNEPWVISVMGYGNGIFAPGH VSNTEPWIVSHHIILAHAHAVKLYRDEFKEKQGGQIGIT LDSHWLIPYDDTDASKEATLRAMEFKLGRFANPIYKGE YPPRIKKILGDRLPEFTPEEIELVKGSSDFFGLNTYTTHL VQDGGSDELAGFVKTGHTRADGTQLGTQSDMGWLQT YGPGFRWLLNYLWKAYDKPVYVTENGFPVKGENDLP

		VEQAVDDTDRQAYYRDYTEALLQAVTEDGADVRGYF GWSLLDNFEWAEGYKVRFGVTHVDYETQKRTPKKSA EFLSRWFKEHIEE
SdegBgl-1	Q21EM1	MKTFNPDFVWGAASSAYQVEGATTTDGRGPSIWDAFS SIPGKTYHNQNADIACDHYNRWQEDVAIMKEMGLKA YRFSISWSRIFPTGRGEVNEKGVAFYNNLIDELIKNDITP WVTLFHWDFPLALQMEMDGLLNPAIADEFANYAKLC FARFGDRVTHWITLNEPWCSAMLGHGMGSKAPGRVS KDEPYIAAHNLLRAHGKMVDIYRREFQPTQKGMIGIA NNCDWREPKTDSELDKKAAERALEFFVSWFADPIYLG DYPASMRERLGERLPTFSDEDIALIKNSSDFFGLNHYTT MLAEQTHEGDVVEDTIRGNGGISEDQMVTLSKDPSWE QTDMEWSIVPWGCKKLLIWLSERYNYPDIYITENGCAL PDEDDVNIAINDTRRVDFYRGYIDACHQAIEAGVKLKG YFAWTLMDNYEWEEGYTKRFGLNHVDFTTGKRTPKQ SAIWYSTLIKDGGF
HsapCyBgl	Q9H227	MAFPAGFGWAAATAAYQVEGGWDADGKGPCVWDTF THQGGERVFKNQTGDVACGSYTLWEEDLKCIKQLGLT HYRFSLSWSRLLPDGTTGFINQKGIDYYNKIIDDLLKNG VTPIVTLYHFDLPQTLEDQGGWLSEAIIESFDKYAQFCF STFGDRVKQWITINEANVLSVMSYDLGMFPPGIPHFGT GGYQAAHNLIKAHARSWHSYDSLFRKKQKGMVSLSL FAVWLEPADPNSVSDQEAAKRAITFHLDLFAKPIFIDG DYPEVVKSQIASMSQKQGYPSSRLPEFTEEEKKMIKGT ADFFAVQYYTTRLIKYQENKKGELGILQDAEIEFFPDPS WKNVDWIYVVPWGVCKLLKYIKDTYNNPVIYITENGF PQSDPAPLDDTQRWEYFRQTFQELFKAIQLDKVNLQV YCAWSLLDNFEWNQGYSSRFGLFHVDFEDPARPRVPY TSAKEYAKIIRNNGLEAHL
RratCyBgl		MTVYKGGWDADGRGPCVWDTFTHQGGERVFENQTG DVACGSYTLWEEDLKCIKQLGLTHYRFSLSWSRLLPD GTTGFINQKGIDYYNKIIDDLLRNGVTPIVAIYHFDLPQ ALEDLGGWLSEAIVEAFDKYAQFCFSTFGDRVKQWLT INEPNILALLAYDMGIFAPGVPHIGIGGYQAAHNLIKAH ARSWHSYDSLFREEQKGFVSLSLFFCWLEPADPNSAID QEATKRAINFHLDFFAKPIFIDGDYPDVVKSQVASMSK KQGYPSSRLPEFTEEEKKMIKGTADFFAVQYYTTRLVR HQDNKKRELGFLQDVEIEFFPNPFWKNVGWIYVVPWG IRKLLKYIKDTYNNPVIYITENGFPQCDPPSLDDTQRWE YFRQTFQELFKAIHVDDVNLQLYCAWSLLDNFEWNNG YSRRFGLFHVDFEDPARPRTPYTSAKEYAKVIRNNGLA GAM
CcanCyBgl	A0A8B7TQ98	MAFPVGFGWGAATAAYQVEGGWDADGRGPCVWDTF THQGGDRVFKNQTGDVACGSYTLWEEDLKCIKQLGLT HYRFSLSWSRLLPDGTTGFINQKGIDYYNKIIDDLLANG VKPIVAIYHFDLPQALEDQGGWLSEAIIEVFDKYSQFCF

		STFGDRVKQWITINEPNTLATMAYDFGIFAPGVPHIGT GGYQAAHNMIKAHAKSWHSYDSLFRKEQKGMVSLSL FVCWLEPADPNSKPDQEAAKRAINFQLDFFAKPIFIDG DYPELVKSQIAYMSKKQGYPSSRLPEFTEEEKKMIKGT ADFFAVQYYTSRLVKHQESNKGELGFLQDVGIEYFPDP SWKGVGWIYVVPWGIRKLLKYIKDMYNSPVIYITENG FPQCDPPSLDDTQRWEYFRQTFQELFKAIHVDKVNLQL YCAWSLLDNFEWNNGYSRRFGLFHVDFEDPARPRVPY RSAKEYAKIIKSNGLEGPL
CporCyBgl	P97265	MAFPADLVGGLPTAAYQVEGGWDADGRGPCVWDTF THQGGERVFKNQTGDVACGSYTLWEEDLKCIKQLGLT HYRFSISWSRLLPDGTTGFINQKGVDYYNKIIDDLLTNG VTPVVTLYHFDLPQALEDQGGWLSEAIIEVFDKYAQFC FSTFGNRVRQWITINEPNVLCAMGYDLGFFAPGVSQIG TGGYQAAHNMIKAHARAWHSYDSLFREKQKGMVSLS LFCIWPQPENPNSVLDQKAAERAINFQFDFFAKPIFIDG DYPELVKSQIASMSEKQGYPSSRLSKFTEEEKKMIKGT ADFFAVQYYTTRFIRHKENKEAELGILQDAEIELFSDPS WKGVGWVRVVPWGIRKLLNYIKDTYNNPVIYITENGF PQDDPPSIDDTQRWECFRQTFEELFKAIHVDKVNLQLY CAWSLLDNFEWNDGYSKRFGLFHVDFEDPAKPRVPYT SAKEYAKIIRNNGLERPQ
OpriCyBgl		MAFPAGFGWGAGTAAYQIEGGWDADGRGPCVWDTF THQGGDRIFKNQTGDVACNSYTLWEEDLKCIKQLGLT HYRFSLSWSRLLPDGTTGFINQKGVDYYNKIIDDLLKN KIIPIVTLFHFDLPQALEDRGGWLSEATIDIFDQYACFCF RTFGDRVKHWITINEANGFAILTYDLGFFAPGVPHIGTG GYQAAHNLIKAHARAWHSYNSLFRKEQKGLVSLSFFS VWLEPADPNSASDKKASERALAFELGTFAKPIFIDGDY PEVVKSQVASMSQRQGYPSSRLPEFTEEEKKMIKGTAD FFAIQYYTTRLIKHKENKKGELGFLQDVEIDCSTDPSW KGENWVCVVPWGLRKLLKHVKDTYNNPVIYITENGFP QRDPPSLDDTQRWECFRQTFQELSKAIQVDKVNVQVY CAWSLLDNFEWNDGYNTRFGLYHVDFEDPARPRVPY TSAKEYAKVIRNNGLEEKP
CasinPRI	A0A2R6RAC3	MAQISSFNRTSFPDGFVFGIASSAYQFEGAAKEGGKGP NIWDTFTHEFPGKISNGSTGDVADDFYHRYKEDVKVL KFIGLDGFRMSISWARVLPRGKLSGGVNKEGIAFYNNV INDLLSKGIQPFITIFHWDLPQALEDEYGGFLSPHIVNDF RDFAELCFKEFGDRVKHRITMNEPWSYSYGGYDAGLL APGRCSAFMAFCPKGNSGTEPYIVTHNLLLSHAAAVKL YKEKYQAYQKGQIGITLVTYWMIPYSNSKADKDAAQR ALDFMLGWFIEPLSFGEYPKSMRRLVGKRLPRFTKEQA MLVKGSFDFLGLNYYIANYVLNVPTSNSVNLSYTTDSL SNQTAFRNGVAIGRPTGVPAFFMYPKGLKDLLVYTKE KYNDPVIYITENGMGDNNNVTTEDGIKDPQRVYFYNQ

		HLLSLKNAIAAGVKVKGYFTWALLDNFEWLSGYTQRF GIVYVDFKDGLKRYPKDSALWFKK
CcelBgl	B8I5U2	MAFKEGFVWGTATASYQIEGAVNEGGRGESVWDEFC RMKGKIDDDDNGDSACDSYHRYSEDIQLMKEIGIKAY RFSISWTRILPDGIGEINMEGVNYYNNLINGLLENGIEP YVTLFHWDYPMELQYKGGWLNPESPLWFENYAAICS RLFSDRVKYWITSNESQCYIGFGYGTGWHAPGFKLPV NQVVRAWHHNLKGLGLAAKAIRENAKGEVKVGLVA CGEVGIPASDSEADMQAARNVLFDREHSEDSIDFGYGD LFEPALKGEYPKSLIPYLPKGWQEDMKDICVPLDFLGV NAYIGSIVEACENKKYRHLKLPVGIGKTSMEWPFKPET LYWVTRFISERYKLPVYITENGMANNDWISTDGKINDT QREDYLNQYLSALSKSIDDGADVRGYFYWSLLDNFEW AYGYAKRFGLVYVDYSNFSRTLKQSALRYKKIIELNGE VLK
CrumBgl-8		MAKYDFPKDFNWGTATASYQVEGGAHEDGKGPSIWT EFEKRPGAIFNGDNGDVASDQYHHWKEDIELMKYLGL RSYRFSMAWSRVIPEGRGAINVAGLDYYKRLCDALLE NGIEPYMTFYHWDLPLALQKEFGGWESRETVKYFGEY VERISKELKGRVKNYFTTNEFLACSDVGYGMGSIAPGL KLPAKRLNQVRHHVLLAHGTALAALRATSPEAKVGLA ENPWFMVPLIDTPEHVEATKLAFREENAHFLTAIMEGK YLDCYLEKCGADAPEFTDDDMKIIGGKVDLLGLNIYFG KYVCKEDDKPYRIFRDDIQSTKAGRPGLYYEPDAIYW GARIVTELWNVPELIVSENGTAMPEDNIDVDSGRVYDL GRIKYLRNYLTSMARAISEGYPIKGYFHWSLVDNLEW NQGLQPRFGLTYIDFHTLKRTLKMSGEWYRELIRTGRI V
CrumBgl-1		MVQFPADFTWGVACASYQCEGGWNADGKGPSIWDDF CHELNGHHVKNDDSGDVACDSYHRYREDVALMKAH NIRAYRFSISWPRVIPDGDGAVNEAGLAYYDALVDLLL ENGIEPMVTLYHWDLPSALQHRGGWQNRQIADWFAR YADIIARRFAGRVKRYMTINEAQCITELGYGRGVLAPG LQLPDEELARIYHNIALAHSAAQRAIKAVSPDAVVGFV SCGKFCFPEHDTPEAVDAAYRAMFEMDEGWGFNFNV VLDSLILRRWDDSAPAAVRRFVETIPPEDWDLMEAPDF VGINVYNGGMVDDAGKPVPHVPGHPITACKWPITPRV MRYGPLLIHRRYGLPMIITENGLSCNDIRFMDGQVHDL KRIDFLHRYLTELSKAIADGAPVLGYLQWSFLDNFEW ASGYDERFGIIYVDYQTLERTPKDSARWYAKVIETNGA CLN
CrumBgl-4		MNFPKDFLWGVATSSYQIEGAEHEDGRCKSVWDDFY KIPRKVVDEKSGAIACDHYHRYKEDVQLIKNLGVKAY RFSVAWPRIFSYDSDSRNGVVKGNLNQKGLDFYDRLI DELLQNGIEPWLTLFHWDLPYELEKKGGWRNRDIHH WISDYSAEIARRYSDRVTHFFTLNEMPCILGGYRGWFA

		PGLEVNEREVFNIIHHMLLSHGSMVQAVRANAKQNVL LGCAHNGLGHYPASESKEDYEAFIKAMNCIEAAPGRY APQEGSGILSGDSLTYYLDPIHFGKYPDKAFELFADKM PEIKDGDMKLISSPVDYQGINIYEGRPITAGSAPGKKDG GWHIEPFEEGYNITAAKWPITPKSMNHYFKFISDRYKK PVYVSENGMSNADIVSLDGKCHDPQRIDFTERYLAELK KAIDSGADVKGYFHWSLMDNYEWRNGYTERFGLVHV DYQTQKRTPKDSYWWYKELVEKYK
CrumBgl-5		MSFRKDFAWGAATAAFQIEGAWNEDGKSPSIWDVFCT QPGKIEDKSDGTVACDHYHRYKEDVKLMSELGLKAY RFSIAWPRVIPDGRGKVNEKALDFYSNLVDELLKYNIT PYVTLYHWDLPYCLYLKGGWMNPEISDMFEEYTRAV AKRLGDRVKHYITFNEPSVFLGCGCLEGSHAPGHKMG TRDLLNMGHNVLLSHGKAVRALRELVPDAEVGITLAT MPAIPVAKKNEEEAYESYFYCDKNTFVWSDAFWVDPI VLGKYPEKLLSECKDIFPAFTDEDMKLISQKIDFLGQNI YQGRYVGEWKRPAGTAHTELSWDVFDDALEWGIKHF TKRYRLPMYITENGLSCHDWVSLDGKVHDPNRIDFLH RYLRGLKKAAESGCDVRGYFQWSLMDNFEWAKGYN PRFGMIFCDYTTQKRIPKDSAYWYKEVIETNGENL
TnonBgl		MTENAEKFLWGVATSAYQIEGATQEDGRGPSIWDTFA RRPGAIRDGSTGEPACDHYHRYEEDIALMQSLGVGVY RFSVAWPRILPEGRGRINPKGLAFYDRLVDRLLAAGITP FLTLYHWDLPQALEDRGGWRSRETAFAFAEYAEAVA RALADRVPFFATLNEPWCSAFLGHWTGEHAPGLRNLE AALRAAHHLLLGHGLAVEALRAAGARRVGIVLNFAPA YGEDPEAVDVADRYHNRYFLDPILGRGYPESPFQDPPP APILSRDLEAIARPLDFLGVNYYAPVRVAPGTGPLPVR YLPPEGPVTAMGWEVYPEGLYHLLKRLGREVPWPLYI TENGAAYPDLWTGEAVVEDPERVAYLEAHVEAALRA REEGVDLRGYFVWSLMDNFEWAFGYTRRFGLYYVDF PSQRRIPKRSALWYRERIARAQTGGSAH
TcurBgl	D1A786	MAFTADFRWGVATAAYQIEGAVTEDGRGASVWDTFC HESGRIAGGHTGDVACDHYHRWPEDLALMADLGVDA YRFSIAWPRVQPGGRGPANPKGLDFYERLVDGLLERGI TPFVTLFHWDLPQALEDAGGWLSRDTAHRFADYAAL VAGRLGDRVEHWITLNEPVVVTAYGYAFGVYAPGRT LLLDALPTAHHQLLGHGLAVAALREHGRRQKIGLANH YSPAWAQDESSPADRRAAQIFDLFMNRLFTDPVLHGT LPDLSALGGPDPASYVRDGDLAAIAAPIDFLGVNYYQP TRLQAPPAGGPLPFEIVPITGHPVTGMGWPVVPDALLS LLRDLRRTHGDALPPILITENGCSYDDAPGPDGTVDDP ERIDFLRAHLQAVETALAEGIDVRGYFVWSLMDNFEW SEGYGPRFGLVHIDYDTQRRTPKTSFAWYRDHIARARR TS

TbisBgl	D6Y5B2	MTAAEQRPLAPGAFPEGFVWGTATSAYQIEGAVDADG RGPSIWDVFCRVPGAIARGESGDHACDHYHRWREDVA LMSELGVGAYRFSVAWPRVLPEGAGRVEQRGLDFYR RLVDELRARDIEPFVTLYHWDLPQALEDRGGWRVRDT AERFADYAEVVAGALGDRVRYWITLNEPYCSAIAGYA EGRHAPGAREGHGALAAAHHLLLGHGLATERLRGRP GLRVGITLNMSPAVPAGPAPEDAAAARRMDLLVNRQF TDPLLGRRYPEDMAETFGAITDFSFRREGDLEIIGAPLD FLGVNYYYRIHAAAAPYEQPDPARRTAADIGARTVVP EGVRTSGLGWPVEPEGLHQTLTWLARRYPGLPPIYITE NGYGDDGTLQDDGRIAYLRDHLAALADAIADGVDVR GWFCWSLLDNFEWARGYAARFGLVHVDYATQARTPK ASFHWLRAFLREHAPAGPDQRSGSPSSTR
DdesBgl	C1CXP6,	MTLTRKDFPNGFIFGTATSSYQIEGAASEDGRGPSIWDT FCRQPGRIQDGTSGDVACDHYHLWPEDLDLLRELGVD AYRFSLAWPRIQPSGSGAVNEKGLEFYDRLVDGLLER GIQPYATLYHWDLPQPLQDIGGWANREVAHHFADYA ALVAGRLGDRVRSIATLNEPWCSSFLSYDIGEHAPGLR DRRLALAAAHHLLLGHGQAVQAMRALGKPAELGLVL NLTPAYPASQSAEDARATQYADGYANRWFLDPVFRG AYPQDMWDAFGQDVPDVQDGDLALIREPLDFLGVNY YTRSLVSAQGPVRPQDAEYTHMHWEVYPQGLTDLLL RLQREYPVPPMYITENGAAYPDERGHADIVHDPERLA YYQRHLAAVIEATRQGADVRGYFAWSMLDNFEWAY GYSRRFGLFYVDYQTQERTWKDSGRWFQGLMARTPV AAD
CflaBgl	D5ULE7	MTSTTRPSGRAFPADFLWGSATASYQIEGAVAEDGRA PSIWDTFSHTPGKVLDGDTGDVAVDHYHRVPQDVAIM QDLGLQAYRFSISWSRVLPAGTGEVNQAGLDFYSDLV DRLIAADIKPVVTLYHWDLPQTLEDAGGWTNRATAEA FAAYARVVARALGDRVHLWTTLNEPWCSAFLGYGSG VHAPGVTDPAAALAAVHHLNLAHGLAATAIREELGAA TPVSITLNLHVTRAASPAPADVEAKRRIDTIANEVFLGP LLEGAYPERVFADTAAISDWSFVQEGDLELIRVPIDLLG VNYYSTGRVQHGTPPVGDGTPGPDGHRSSVVSPWIGA DNVEWLPQPGPHTAMGWNIEPQGLVDLLLELHERYPE LPLAITENGAAFYDTVTDDGRVHDPDRVAYLHDHVDA VGEARDKGVDVRGYFVWSLFDNFEWAYGYDRRFGV VHVDYDTQVRTLKDSARWYRELVRTGTIPTPESAASL
BbreBgl	P94248	MTMIFPKGFMFGTATAAYQIEGAVAEGGRTPSIWDTFS HTGHTLNGDTGDVADDFYHRWEDDLKLLRDLGVNAY RFSIGIPRVIPTPDGKPNQEGLDFYSRIVDRLLEYGIAPIV TLYHWDLPQYMASGDGREGGWLERETAYRIADYAGI VAKCLGDRVHTYTTLNEPWCSAHLSYGGTEHAPGLG AGPLAFRAAHHLNLAHGLMCEAVRAEAGAKPGLSVT LNLQICRGDADAVHRVDLIGNRVFLDPMLRGRYPDEL

		FSITKGICDWGFVCDGDLDLIHQPIDVLGLNYYSTNLV KMSDRPQFPQSTEASTAPGASDVDWLPTAGPHTEMG WNIDPDALYETLVRLNDNYPGMPLVVTENGMACPDK VEVGTDGVKMVHDNDRIDYLRRHLEAVYRAIEEGTD VRGYFAWSLMDNFEWAFGYSKRFGLTYVDYESQERV KKDSFDWYRRFIADHSAR
TfusBgl		MTSQSTTPLGNLEETPKPDIRFPSDFVWGVATASFQIEG STTADGRGPSIWDTFCATPGKVENGDTGDPACDHYNR YRDDVALMRELGVGAYRFSIAWPRIQPEGKGTPVEAG LDFYDRLVDCLLEAGIEPWPTLYHWDLPQALEDAGG WPNRDTAKRFADYAEIVYRRLGDRITNWNTLNEPWCS AFLGYASGVHAPGRQEPAAALAAAHHLMLGHGLAAA VMRDLAGQAGRSVRIGVAHNQTTVRPYTDSEADRDA ARRIDALRNRIFTEPLVKGRYPEDLIEDVAAVTDYSFV QDGDLKTISANLDMMGVNFYNPSWVSGNRENGGSDR LPDEGYSPSVGSEHVVEVDPGLPVTAMGWPIDPTGLY DTLTRLANDYPGLPLYITENGAAFEDKVVDGAVHDTE RIAYLDSHLRAAHAAIEAGVPLKGYFAWSFMDNFEWA LGYGKRFGIVHVDYESQTRTVKDSGWWYSRVMRNGG IFGQE
TterBgl	D1CGH4	MSQPRTDLAPGRFPADFTWGTATAAYQIEGAVREDGR GVSIWDRFSHTPGKTHNGDTGDVACDHYHRWQGDIE LMRRLHVNAYRFSIAWPRILPEGWGRVNPPGLDFYDR LVDGLLAAGITPWVTLYHWDLPQALEDRGGWPNPDT SKAFAEYADVVTRRLGDRVKHWITLNEPWVVAFLGY FTGEHAPGRKEPESYLPVVHNLLLAHGLAVPVIRENSR DSQVGITLNLTHAYPAGDSAEDEAAAKRLDGFMNRW FLDPLFTGGYPRDMIDVFGSWVPSFDESDLGVIGAPLD FLGVNYYSPSFVQHSEGNPPLHVEQVRVDGEYTDMG WLVYPQGLYDLLTRLHRDYSPAAIVITENGAAYPDEPP VEGRVHDPKRVEYYASHLDAAQRAIRDGVPLRGYFA WSLMDNFEWAFGYSKRFGLYYVDYETLERTIKDSGL WYSRVVAEGQLVPTESVA
SdegBgl-2	Q21KX3	MNRLTLPPSSRLRSKEFTFGVATSSYQIEGGIDSRLPCN WDTFCEQPNTIIDNTNGAIACDHINRWQDDIELIANLG VDAYRFSIAWGRVINLDGSLNNEGVTFYKNILTKLREK NLKAYITLYHWDLPQHLEDAGGWLNRDTAYKFRDYV NLITQALDDDVFCYTTLNEPFCSAYLGYEIGVHAPGIK DLASGRKAAHHLLLAHGLAMQVLRKNCPNSLSGIVLN MSPCYAGSNAQADIDAAKRADDLLFQWYAQPLLTGC YPDAINSLPDNAKPPICEGDMALISQPLDYLGLNYYTR AVFFADGNGGFTEQVPEGVELTDMGWEVYPQGLTDL LIDLNQRYTLPPLLITENGAAMVDELVNGEVNDIARIN YFQTHLQAVHNAIEQGVDVRGYFAWSLMDNFEWALG YSKRFGITYVDYQTQKRTLKASGHAFAEFVSSRS

VvulBgl	Q7MG41	MNKYQLPQDSQLRQADFLFGVATSSYQIEGGAQLGGR<br>TPSIWDTFCNQPGAVDNMDNGDVACDHFHLWQQDIE<br>LIQGLGVDAYRLSMAWPRILPKDGQVNQQGLEFYERII<br>DECHARGLKVFVTLYHWDLPQYLEDKGGWLNRETAY<br>KFAEYAEVVSGYFGNKIDSYATLNEPFCSAYLGYRWGI<br>HAPGKKGEREGFLSAHHLMLAHGLAMPIMRKNAPQS<br>MHGCVFNATPAYPYSEQDVAAAEYSDAEGFHWFIDPV<br>LKGEYPQSVLEHQAHNMPMILDGDLDIIRGDLDFIGINF<br>YTRCVVRFDANGELESMPQPDAEHTYIGWEIYPQALT<br>DLLLRLKQRYPNLPPVYITENGAAGEDACINGEVNDEQ<br>RVRYFQSHLLALDEAIRAGVNVQGYFAWSLMDNFEW<br>AYGYKQRFGIVHVDYATQKRTLKQSAIAYRNTLLARA<br>EEKQ
CrumBgl-3		MFTRPDLPKDFLIGAATASYQVEGAANEDGRTSCIWD<br>DFAKVPGKVFQCQDGSVAADQYHRYKEDIELMAKLG<br>FKAYRFSVSWSRVLPNGGKKVNPKGIEYYRNLCIELHK<br>HNMKACCTIYHWDMPSEIQAKGGWSNRQTSYELAYL<br>AKVLFEELGDLVDMWITINEAMCITVLGYLLGIHAPGI<br>KDKNQFIRSVHHVNLAHGLVLQEYRKSGLKAPIGITHN<br>LETPRPASKDEKDRLAVQHHIALRDGIFMDPIFKKAYP<br>TYMTDELGWVFPIEDGDFELISQPMDFLGINYYSEHVIT<br>WSDTEPFNVKEVPRWEEKMTGIGWCITPHGLLRLLKW<br>VTEYTNSTIPIYITENGCCSADKLETDPVTKQERVHDTQ<br>RVRYLSDHLNICAEAIKNNIPLKGYFCWSFIDNYEWTY<br>GYSMRFGLVYCDYQTQRRIPKDSAYFMRDVMAGYGD
HoreBgl	B8CYA8	MAKIIFPEDFIWGAATSSYQIEGAFNEDGKGESIWDRFS<br>HTPGKIENGDTGDIACDHYHLYREDIELMKEIGIRSYRF<br>STSWPRILPEGKGRVNQKGLDFYKRLVDNLLKANIRP<br>MITLYHWDLPQALQDKGGWTNRDTAKYFAEYARLMF<br>EEFNGLVDLWVTHNEPWVVAFEGHAFGNHAPGTKDF<br>KTALQVAHHLLLSHGMAVDIFREEDLPGEIGITLNLTP<br>AYPAGDSEKDVKAASLLDDYINAWFLSPVFKGSYPEE<br>LHHIYEQNLGAFTTQPGDMDIISRDIDFLGINYYSRMVV<br>RHKPGDNLFNAEVVKMEDRPSTEMGWEIYPQGLYDIL<br>VRVNKEYTDKPLYITENGAAFDDKLTEEGKIHDEKRIN<br>YLGDHFKQAYKALKDGVPLRGYYVWSLMDNFEWAY<br>GYSKRFGLIYVDYENGNRRFLKDSALWYREVIEKGQV<br>EAN
CtheBgl	P26208	MSKITFPKDFIWGSATAAYQIEGAYNEDGKGESIWDRF<br>SHTPGNIADGHTGDVACDHYHRYEEDIKIMKEIGIKSY<br>RFSISWPRIFPEGTGKLNQKGLDFYKRLTNLLLENGIMP<br>AITLYHWDLPQKLQDKGGWKNRDTTDYFTEYSEVIFK<br>NLGDIVPIWFTHNEPGVVSLLGHFLGIHAPGIKDLRTSL<br>EVSHNLLLSHGKAVKLFREMNIDAQIGIALNLSYHYPA<br>SEKAEDIEAAELSFSLAGRWYLDPVLKGRYPENALKLY<br>KKKGIELSFPEDDLKLISQPIDFIAFNNYSSEFIKYDPSSE

		SGFSPANSILEKFEKTDMGWIIYPEGLYDLLMLLDRDY<br>GKPNIVISENGAAFKDEIGSNGKIEDTKRIQYLKDYLTQ<br>AHRAIQDGVNLKAYYLWSLLDNFEWAYGYNKRFGIV<br>HVNFDTLERKIKDSGYWYKEVIKNNGF
BacGBgl	A0A1I0ZQD8_<br>9BACL	MASIQFPKDFVWGTATASYQIEGAYNEDGRGMSIWDT<br>FSRTPGKVVNGDTGDIACDSYHRYEEDIALLKNLGVK<br>AYRFSIAWPRIYPDGDGELNQKGLDYYAKVIDGLLAA<br>GIEPCVTLYHWDLPQALQDKGGWDNRDTIRAFVRYAE<br>TAFKAFGGKVKQWITFNETWCVSFLSNYIGAHAPGNT<br>DLQLAVNVAHNCMVAHGEAVKAFRALGISGEIGTTHN<br>LYWFEPYTTKPEDVAAAHRNRAYNNEWFMDPTFKGQ<br>YPQFMVDWFKGKGVEVPIQPGDMETIAQPIDFIGVNFY<br>SGGFGRYKEGEGLFDCEEVQVGFDKTFMDWNVYAEG<br>LYKVLSWVHEEYGDVPIYITENGACYEDELTQEGRVH<br>DAKRADYFKKHFIQCHRLIESGVPLKGYFAWSLLDNFE<br>WAEGYVKRFGIVYTDYKTLKRYPKDSYRFIQSVIENDG<br>FEA
BhalBgl	Q9KBK3	MSIIQFPKEMKWGVATASYQIEGAINAGGRGASIWDVF<br>AKTPGKVKNGDNGDVACDSYHRYEEDIEIMKDLGVD<br>MYRFSVAWPRIFPNGTGEVSREGLDYYHRLVDRLTEN<br>GIQPMCTLYHWDLPQALQEKGGWDNRDTIDAFVRYA<br>EVMFKEFGDKINHWITFNELWCVSFLSNYIGVHAPGNT<br>DLQLATNVAHHLLVAHGKAVQSYRKMGLDGQIGYAP<br>NVEWNEPFSNQMEDAEACKRGNGWFIEWFMDPVFKG<br>AYPSFLVEWFEKKGITVPIEAGDMETIQQPIDFLGINYY<br>TGSVARYKENEGLFDLEKVDAGYEKTDIGWNIYPEGF<br>YKVLYYITEQYGQIPIYITENGSCYNDEPVNGQVKDEG<br>RIRYLSQHLTALKRSMESGVNIKGYMAWSLLDNFEWA<br>EGYSMRFGIVHVNYRTLERTKKDSFYWYKQMIANQFF<br>EL
CtroEXG	C5ME42	MISNPSKSNGVKFKRGGNVAWDYENDIVRGVNLGGW<br>FVLEPYMNPSLFEPFKNGNDESGVPVDEYHWTQTLGK<br>ETASKILEDHWAKWITEWDFQQMSNLGLNLVRIPIGY<br>WAFQLLDNDPYVQGQVAFLDEALEWARNHNIKVWID<br>LHGAPGSQNGFDNSGLRDSLEFQNGDNTQVTLNVLAE<br>IFQKYGTSDYDDVVVGIELVNEPLGPSLDMDALKKFY<br>MDGYSSLRNTEGSVTPLIIHDAFQVSGYWDNFLTVAG<br>GQWNVVLDHHHYQVFSAGELSRDIDQHISVACNWGW<br>SAKNEYHWTVTGEWSAALTDCAYWLNGVNRGARWE<br>GAYDGSPYYGSCEPYLQFSSWTDEHKTNVRRYIEAQL<br>DAFEFTGGWIFWSWKTENAIDWDFQKLTDNGIFPQPL<br>DDRQFPNQCGFN
CmalEXG	M3IJY9	MITNPQNNNNNNNVKFKRGGTVAWDYDNDTIRGVNL<br>GGWFVLEPYMNPSLFQPFSSGNGDVGIPLDEYHFTQTL<br>GKDAASEILQKHWSTWITEDDFQQMSSLGLNFARIPIG<br>YWAFELLSNDPYVQGQVEYLDQALEWARNSNIKVWI

		DLHGAPGSQNGFDNSGLRDSLQFQNGDNTQATLNALAKIFQKYGGANYSDVVIGIELLNEPLGPSLDMSALQQFFVEGYWSLRNTDGSVTPVIIHDAFQPFGYWDNFLTVANGEWNVVIDHHHYQVFSPGELSRDINQHISVACNWGWDAKKEYHWNIAGEWSAALTDCATWLNGVGRGARWEGAYDGSQYFGSCQPYLQFETWPEDYKTNVRKYVEAQLDAFEYTGGWVFWSWKTENAIEWDFQKLTANGIFPQPLTDRWYPNQCGFN
AcreRut	A0A286JZ59	MAPQAAYLDWKAFRANGVNLGGWLHQEAVIDPVWWSENGGDGIPDEWGLCAKLGRLCGPRLEQRYASYITTQDIDEMAEAGINVLRIPTGYNAWVKVPGSQLYTGNQVRFLRSISDYAIRKYGMHIIVDIHSAPGGLNGMGLGGREGGYGWFQNETALDYSFRAVDAAIAFIQSSSHPESFTLEPLNEPVDNRNMAEFGTPAALTPEGVAWVLKYFRGVLSRVQKVDARIPVMLQGSFKGEDFWSPYFAATDNIVFDVHHYYFAGRPTTSANLPEWICTDAKGAVGDGVFPVFTGEWSIQAATANTFASRALNLNTGLKVFGEYSRGSAYWTWKFSGNVPVEGEGVQGDYWSYEKFFEAGYINPSEGVSCQ
AniRut	A0A6B9UJ04	MAPLASPPNSSYIDWRTFKGNGVNLGGWLEQESTIDSLFWDKYSGGASDEWGLCEHLGSQCGPVLEHRYATLITKADIDKLASGGITVLRIPTTYAAWIDLPSSQLYSGNQTAYLKEIADYAIKTYNMHIIIDTHSLPGGVNGLTIGEATGHWYWFYNETHFNYSMQVIDQVINFIQTSGSPQSYTLEPINEPADNNTNMVVFGTPLALTDHGAAWVLKYIRAVVQRVESVNPNIPVMFQGSFKYPQYWEGDFPASTNLVFDTHHYYYEHMDSSSENLPEYILADAREKSGTGKFPVFVGEWAIQATYNNTLALRKRNVLAGLETWSSFSQGSSYWTAKFTGNTSVAGQGEQKDYWCYETFIDEGYFN
